# TYRO3 agonist as therapy for glomerular disease

**DOI:** 10.1172/jci.insight.165207

**Published:** 2023-01-10

**Authors:** Fang Zhong, Hong Cai, Jia Fu, Zeguo Sun, Zhengzhe Li, David Bauman, Lois Wang, Bhaskar Das, Kyung Lee, John Cijiang He

**Affiliations:** 1 Department of Medicine/Nephrology, Icahn School of Medicine at Mount Sinai, New York, New York, USA.; 2 Department of Nephrology, Renji Hospital, School of Medicine, Shanghai Jiao Tong University, Shanghai, China.; 3 Arnold and Marie Schwartz College of Pharmacy and Health Sciences, Long Island University, New York, New York, USA.; 4 Renal Section, James J. Peters Veterans Affairs Medical Center, New York, New York, USA.

**Keywords:** Nephrology, Chronic kidney disease

## Abstract

Podocyte injury and loss are key drivers of primary and secondary glomerular diseases, such as focal segmental glomerulosclerosis (FSGS) and diabetic kidney disease (DKD). We previously demonstrated the renoprotective role of protein S (PS) and its cognate tyrosine-protein kinase receptor, TYRO3, in models of FSGS and DKD and that their signaling exerts antiapoptotic and antiinflammatory effects to confer protection against podocyte loss. Among the 3 TAM receptors (TYRO3, AXL, and MER), only TYRO3 expression is largely restricted to podocytes, and glomerular *TYRO3* mRNA expression negatively correlates with human glomerular disease progression. Therefore, we posited that the agonistic PS/TYRO3 signaling could serve as a potential therapeutic approach to attenuate glomerular disease progression. As PS function is not limited to TYRO3-mediated signal transduction but includes its anticoagulant activity, we focused on the development of TYRO3 agonists as an optimal therapeutic approach to glomerular disease. Among the small-molecule TYRO3 agonistic compounds screened, compound 10 (C-10) showed a selective activation of TYRO3 without any effects on AXL or MER. We also confirmed that C-10 directly binds to TYRO3, but not the other receptors. In vivo, C-10 attenuated proteinuria, glomerular injury, and podocyte loss in mouse models of Adriamycin-induced nephropathy and a *db/db* model of type 2 diabetes. Moreover, these renoprotective effects of C-10 were lost in *Tyro3*-knockout mice, indicating that C-10 is a selective agonist of TYRO3 activity that mitigates podocyte injury and glomerular disease. Therefore, C-10, a TYRO3 agonist, could be potentially developed as a new therapy for glomerular disease.

## Introduction

Primary and secondary glomerular diseases are responsible for almost two-thirds of end-stage kidney disease (ESKD) (1), and primary glomerular disease, such as focal segmental glomerulosclerosis (FSGS), is a leading cause of ESKD (1). Diabetic kidney disease (DKD), a secondary glomerular disease, is the most common cause of ESKD in the United States and globally (1). The current treatment regimens for both primary and secondary glomerular diseases are limited. Since podocyte injury and loss is a critical event leading to the development and progression of DKD and FSGS (2–5), treatments specifically targeting podocyte injury and loss would be an important approach to prevent albuminuria and to slow the glomerular disease progression. To develop a potential therapy, we recently focused on identification of molecules that may mediate renoprotection against podocyte injury in experimental models of glomerular disease, and established protein S (PS) as one of these molecules (6). We showed that PS expression is significantly increased in the podocytes during the early stages, but decreased at the late stages of DKD, and that the loss of PS exacerbates podocyte injury and DKD progression of DKD in mice. In contrast, the podocyte-specific overexpression of PS attenuates kidney injury in DKD, supporting a protective role of PS in glomerular disease (6).

PS shares structural similarities with its homolog GAS6, and both PS and GAS6 bind to TAM tyrosine kinase receptors (TYRO3, AXL, and MER receptors) that regulate various biological processes, including cell survival and inflammation (7). Although structurally similar, PS and GAS6 have disparate binding affinities for individual TAM receptors (8, 9), leading to divergent functions (10). GAS6 binds with the strongest affinity to AXL, whereas PS does so to TYRO3 (7, 11). GAS6/AXL signaling induces mesangial cell proliferation and glomerular hypertrophy in early DKD through the activation of the Akt/mTOR pathway (12, 13), thereby promoting diabetic glomerulopathy. In contrast, PS/TYRO3 reduces cellular injury by reducing the inflammatory responses (14–16). Consistent with these observations, we showed that PS has antiinflammatory and antiapoptotic effects through activation of TYRO3 in cultured podocytes (6).

However, independent of TYRO3 activation, PS plays a significant role in the anticoagulation system, and would not be an optimal target for the treatment of glomerular disease. Similar to PS knockout, we previously showed that TYRO3 aggravates podocyte injury, and conversely, the induction of TYRO3 in podocytes attenuates injury in animal models of glomerular disease (17). Therefore, as a potential therapeutic target in glomerular disease, we focused on the agonism of TYRO3. Using medicinal chemistry and in vivo experimental approaches, we demonstrate that select TYRO3 agonism markedly improves kidney function and attenuates podocyte injury in experimental models of DKD and FSGS and that small-molecule TYRO3 agonists, such as compound 10 (C-10) described in this study, can be further developed as specific therapy for glomerular disease.

## Results

### TYRO3 expression is found almost exclusively in podocytes of mouse and human kidneys.

We first determined kidney cell–specific mRNA expression of TYRO3 using the recently published single-cell RNA sequencing (scRNAseq) data of mouse and human kidneys. The mouse glomerular scRNAseq data set (18) showed that while all 3 TAM receptors were expressed in podocytes (Supplemental Figure 1A; supplemental material available online with this article; https://doi.org/10.1172/jci.insight.165207DS1), *Tyro3* expression was limited to podocytes as compared to the broader expression of *Mer* and *Axl*. In another recently published mouse kidney scRNAseq data set, which contains all major kidney cell subtypes but without significant representation of glomerular cells (19), *Tyro3* expression was absent in nonglomerular cells, while *Mer* was broadly expressed and *Axl* was expressed in kidney macrophages (Supplemental Figure 1B). We further examined an scRNAseq data set of CD45-enriched kidney immune cells (20), which similarly showed minimal expression of *Tyro3,* but varying degrees of expression of *Mer* and *Axl* in immune cell subsets (Supplemental Figure 1C). We also examined the expression of *TYRO3* in human kidneys using the single-nucleus RNAseq data sets of control subjects and DKD patients (http://humphreyslab.com/SingleCell) (21). *TYRO3* expression was limited to podocytes in both control and diabetic kidneys (Supplemental Figure 1D) and consistent with our earlier observation, *TYRO3* expression in early DKD was not decreased in comparison with control kidneys. These data indicate that kidney expression of TYRO3 is largely restricted to podocytes, unlike the other 2 TAM receptors.

### Small-molecule C-10 is a specific TYRO3 agonist.

**Figure 1:**
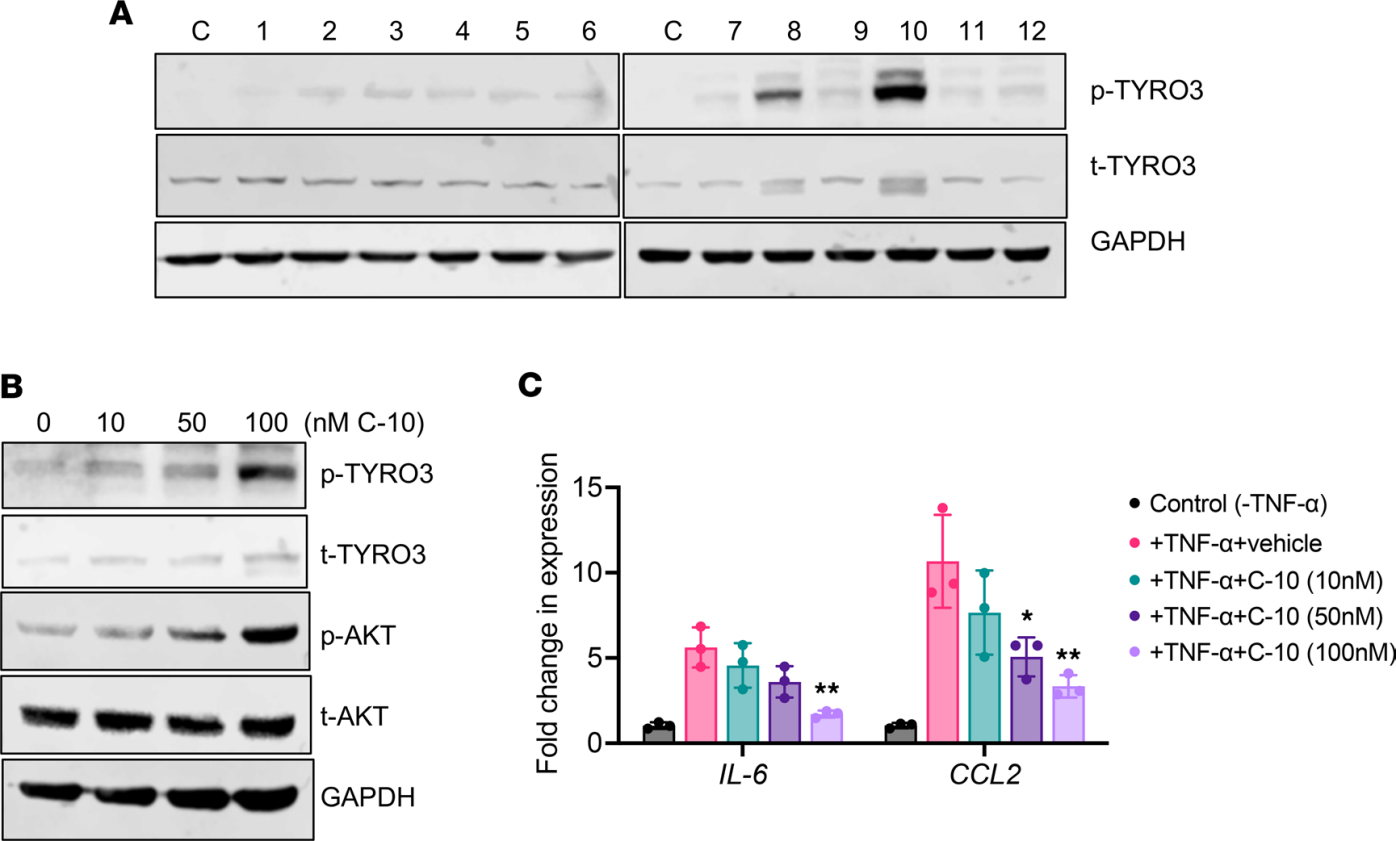
C-10 is a TYRO3 agonist. (**A**) Cultured human podocytes were treated with control vehicle (C) or TYRO3 agonists (compounds 1–12) for 30 minutes. Cell lysates were probed for phosphorylated or total TYRO3 (p-TYRO3 or t-TYRO3). GAPDH was used as a loading control. (**B**) Cultured human podocytes were stimulated with compound 10 (C-10) for 30 minutes at different doses as indicated, and cell lysates were probed for p-TYRO3 or t-TYRO3 and for p-AKT or t-AKT. GAPDH was used as a loading control. (**C**) Cultured podocytes were treated with vehicle control (C), 10 μM TNF-α alone (T), or TNF-α with TYRO3 agonists (compounds 1–12) for 2 hours. Real-time PCR analysis was performed for expression of NF-κB target genes, *IL6* and *CCL2* (*n* = 3). *P < 0.05, **P < 0.01 compared with TNF-α–treated cells by 1-way ANOVA with Bonferroni’s correction.

For the development of a small-molecule agonist of TYRO3, we undertook 2 approaches of in silico homology modeling, in the absence of a known crystal structure of TYRO3, by taking advantage of the specificity of ligand-receptor interactions between PS/TYRO3 and GAS6/AXL. In the first approach, by comparison of the PS structure with that of GAS6, which binds TYRO3 with weak affinity, we deduced the potential allosteric interaction sites between PS and the extracellular region of TYRO3. In the second approach, we compared the tyrosine autophosphorylation sites of TAM receptors in the intracellular region (TYRO3-Y804, AXL-Y821, and MER-Y872) that are surrounded by unique neighboring sequences according to the PDBsum database (22). Based on these 2 approaches, we designed 12 compounds with 3 different pharmacophore groups, shown in Supplemental Figure 2A. Supplemental Figure 2B shows the examples of synthesis reactions of the 2 select compounds. We first screened the 12 compounds based on their effects on TYRO3 phosphorylation, as a surrogate for its increased signaling activity, in cultured human podocytes. As shown in Figure 1A, C-8 and C-10 showed robust phosphorylation of TYRO3 among the screened compounds. Because C-10 and C-8 shared structural similarities (Supplemental Figure 2, A and B), but the effect of C-10 was superior to that of C-8, we focused on further characterization of C-10 in cultured human podocytes. Treatment with C-10 led to dosage-dependent phosphorylation of TYRO3 and activation of its downstream molecule, AKT (Figure 1B). We previously demonstrated that PS/TYRO3 signaling attenuated the NF-κB–mediated inflammatory response in podocytes (6, 17). Therefore, we also examined the effects of C-10 in TNF-α–treated podocytes. Real-time PCR analysis of NF-κB target genes *IL6* and *CCL2* showed a significant attenuation of their expression in C-10 dose–dependent manner in cultured podocytes (Figure 1C and Supplemental Figure 3). These results indicate that C-10 is a potent agonist of TYRO3. Moreover, no cellular cytotoxicity was observed in cultured podocytes at these effective dosages (Supplemental Figure 4). Because increased expression of TYRO3 has been observed in several types of cancers, including gastric cancer, we also tested whether C-10 might influence their proliferation. As shown in Supplemental Figure 5, C-10 had no obvious effects on the proliferative ability of gastric cancer cells, even when incubated with 500 nM C-10, a dose nearly 10-fold higher than the dose range that activates TYRO3 in cultured podocytes.

**Figure 2:**
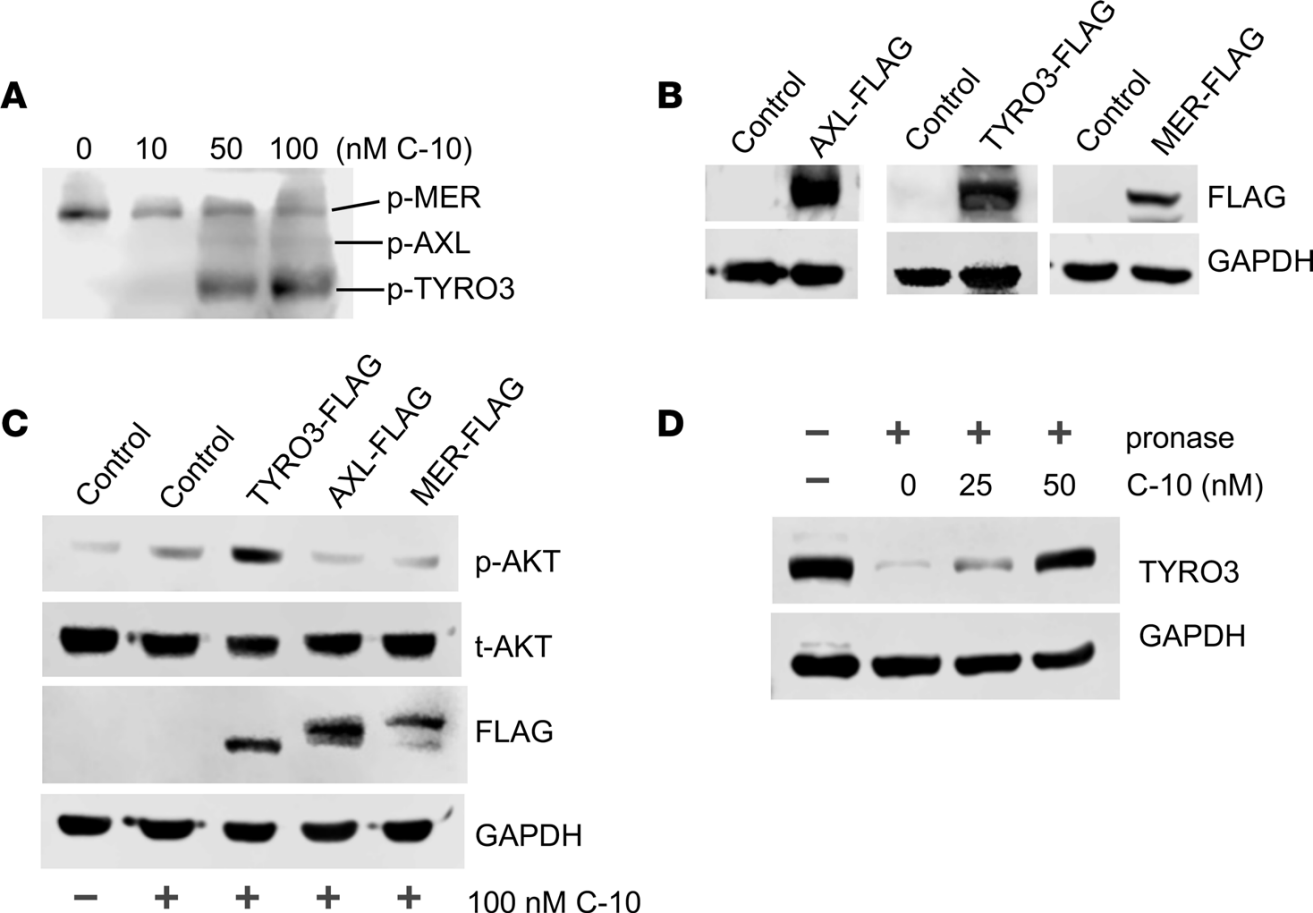
C-10 is a selective agonist of TYRO3. (**A**) Cultured human podocytes were stimulated with C-10 at different doses for 30 minutes. Cell lysates were probed with an antibody recognizing the phosphorylated form of all 3 TAM receptors. Phosphorylated MER, AXL, and TYRO3 receptors are differentiated by their corresponding molecular weight. (**B**) Immortalized podocytes were transduced with either control vector or lentiviral vectors expressing individual FLAG-tagged TAM receptors (AXL-FLAG, TYRO3-FLAG, or MER-FLAG). Lysates from transduced cells were probed for FLAG-tagged protein expression. (**C**) Immortalized podocytes from **B** were treated with 100 nM C-10 for 30 minutes, and lysates were probed for phosphorylated or total AKT (p-AKT or t-AKT) and FLAG proteins. GAPDH was used as a loading control. Representative blots of 3 independent experiments are shown. (**D**) Drug affinity responsive target stability (DARTS) assay was performed to test the direct binding of C-10 to TYRO3. Podocyte lysates were preincubated with various concentrations of C-10 as indicated at 25°C for 1 hour prior to digestion with Pronase (0 or 1:1000 dilution) for 20 minutes. Lysates were then probed for TYRO3 expression, with GAPDH as a loading control.

To test the specificity of C-10 for TYRO3 among the TAM receptors, we next examined the effects of C-10 on the phosphorylation of the endogenous TAM receptors in podocytes. Cultured human podocytes were treated with varying doses of C-10 for 30 minutes, and Western blotting was performed using an antibody that recognizes all phosphorylated TAM receptors. C-10 enhanced TYRO3 phosphorylation in a dose-dependent manner, but it had no effect on AXL and MER activation (Figure 2A). We additionally examined the effects of C-10 in podocytes overexpressing FLAG-tagged TYRO3, AXL, or MER (Figure 2B). C-10 led to a strong induction of AKT phosphorylation only in TYRO3-overexpressing cells (Figure 2C and Supplemental Figure 3). To confirm that C-10 exerts its specific effects on TYRO3 by direct interaction, we utilized the drug affinity responsive target stability (DARTS) method (23–26), which is used to infer direct binding of a small-molecule compound to a protein. It utilizes the change in the target protein conformation resulting from a direct interaction with the small molecule, thereby altering its susceptibility to proteolysis. As shown in Figure 2D, increasing concentrations of C-10 conferred resistance to Pronase-mediated degradation of TYRO3 (Figure 2D and Supplemental Figure 3), but it had no effects on Pronase-mediated AXL or MER degradation (data not shown). Together, these results strongly indicate that C-10 is a potent and select agonist of TYRO3.

### C-10 attenuates glomerular injury in mice with Adriamycin-induced nephropathy.

**Figure 3:**
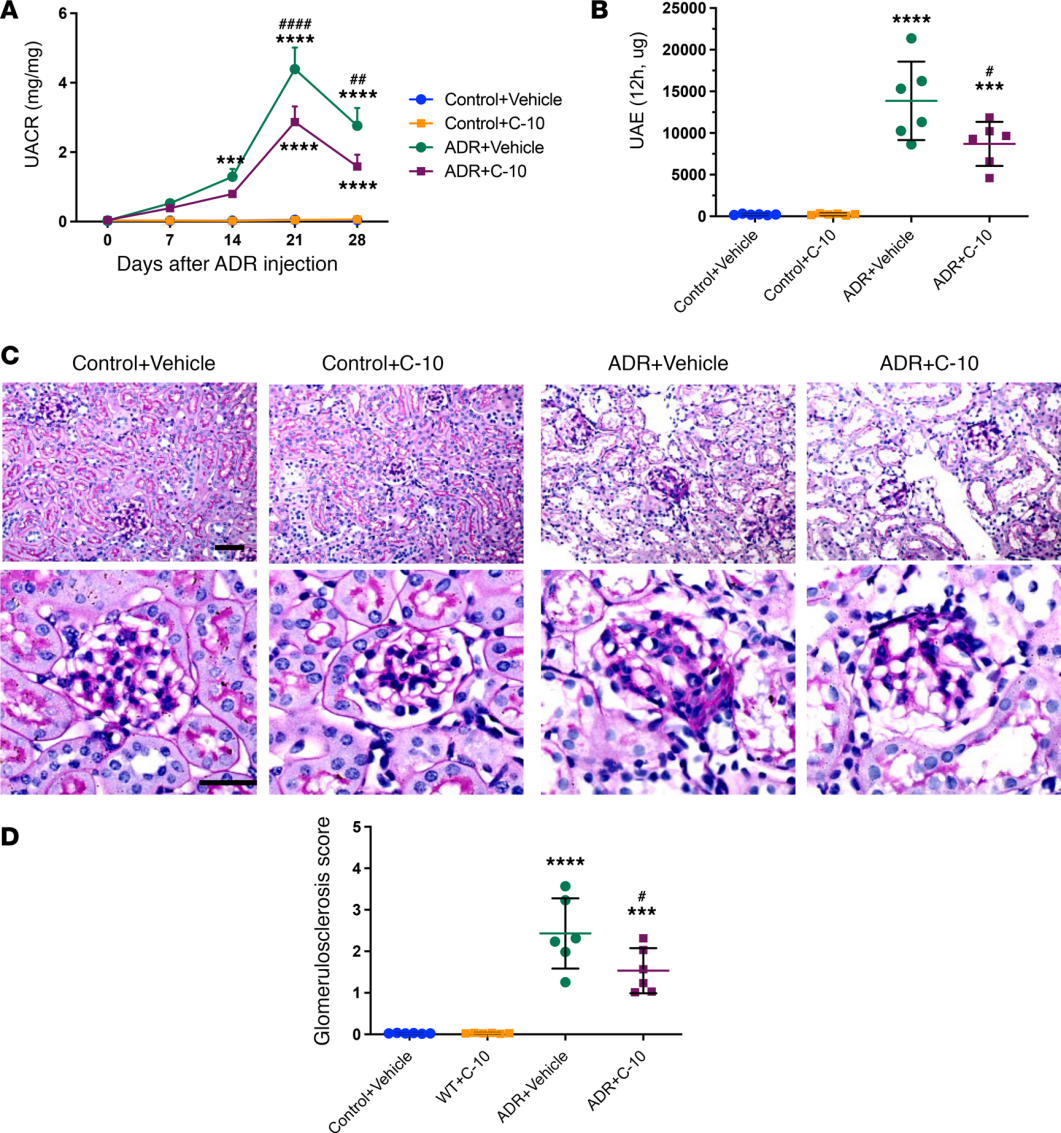
C-10 ameliorates albuminuria and glomerulosclerosis in mice with ADRN. (**A**) Urinary albumin/creatinine ratio (UACR) in control or ADRN mice treated with vehicle or C-10. (**B**) Twelve-hour urinary albumin excretion (UAE) in control and ADRN mice. (**C**) Periodic acid–Schiff–stained kidney images at ×200 (top) and ×400 (bottom) magnifications. Scale bars: 50 μm. (**D**) Glomerulosclerosis scoring in control and ADRN mice. (*n* = 6 mice per group). ***P < 0.001, ****P < 0.0001 compared with control group; ^#^*P* < 0.05, ^##^*P* < 0.01, ^####^*P* < 0.0001 compared with ADR+Vehicle mice by 1-way ANOVA with Bonferroni’s correction.

**Figure 4:**
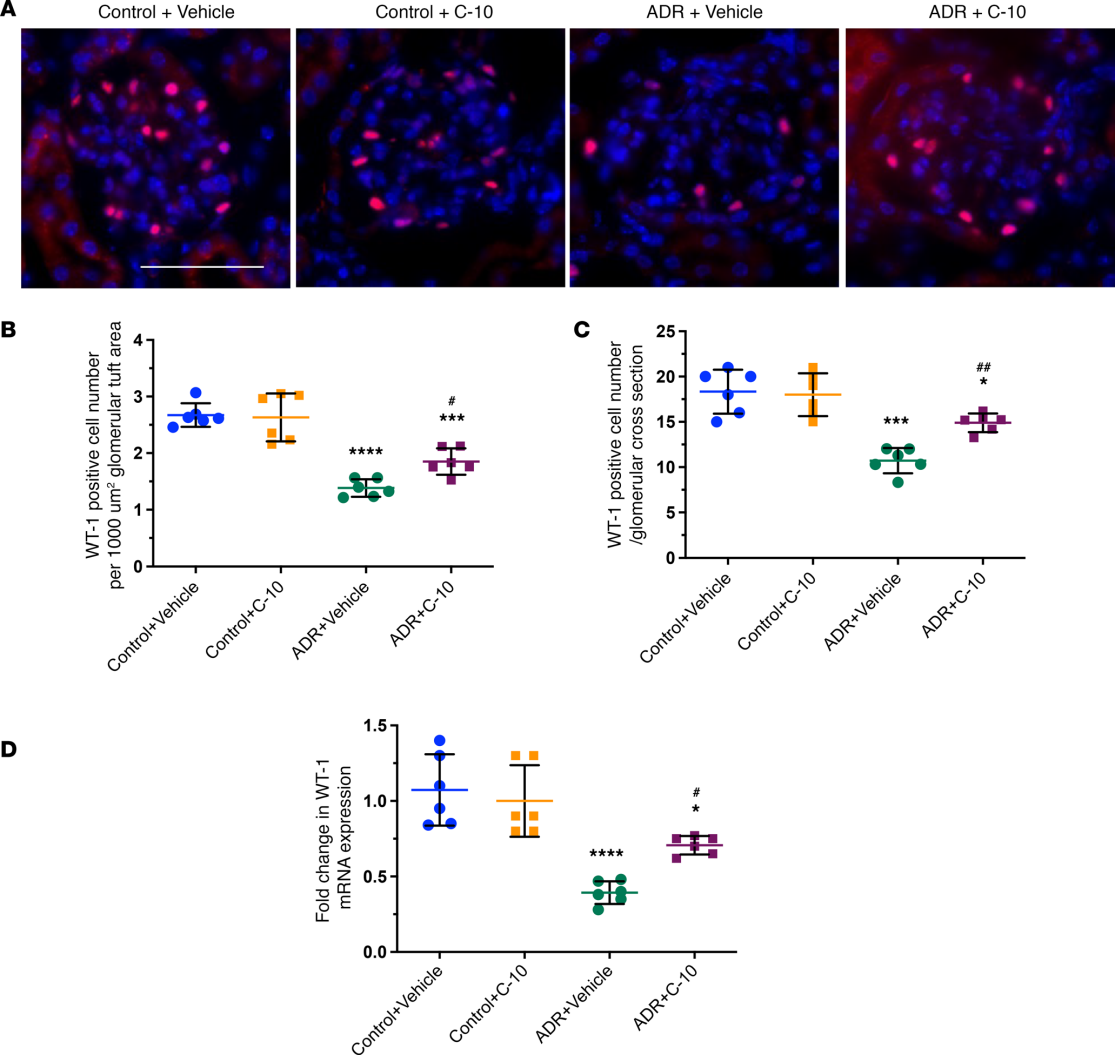
C-10 ameliorates podocyte loss in mice with ADRN. (**A**) Representative images of WT-1 staining in glomeruli are shown (*n* = 6). Original magnification, ×400. Scale bar: 50 μm. (**B** and **C**) WT-1–positive cell number per 1000 μm^2^ glomerular tuft area (**B**) and per glomerular cross section (**C**) were quantified and are expressed as fold changes relative to control mice (60 glomeruli quantified per group). (**D**) mRNA levels of *Wt1* were determined using real-time PCR (*n* = 6 mice per group). *P < 0.05, ***P < 0.001, ****P < 0.0001 compared with control group; ^#^*P* < 0.05, ^##^*P* < 0.01 compared with ADR+Vehicle mice by 1-way ANOVA with Bonferroni’s correction.

We next tested the efficacy of TYRO3 agonism by C-10 in vivo using the experimental model of FSGS, Adriamycin-induced nephropathy (ADRN). ADRN was induced in 10-week-old male BALB/c mice by Adriamycin injection (8 mg/kg body weight). Mice were treated with either vehicle control or C-10 (2.5 mg/kg body weight) by daily intraperitoneal injection, 3 days after Adriamycin administration. Kidneys were examined 4 weeks after Adriamycin administration. C-10 significantly reduced albuminuria in ADRN mice by both spot collection and 12-hour collection of urine samples (Figure 3, A and B). There was also a significant attenuation of glomerulosclerosis development with C-10 treatment (Figure 3C and 3D). C-10 also restored podocyte numbers in ADRN mice, as assessed by Wilms tumor 1 (WT-1) staining (Figure 4, A–D), and reduced ADR-induced podocyte foot process effacement (Supplemental Figure 6). No obvious toxicity was observed in these mice during the treatment period.

### C-10 attenuated glomerular injury in mice with DKD.

**Figure 5:**
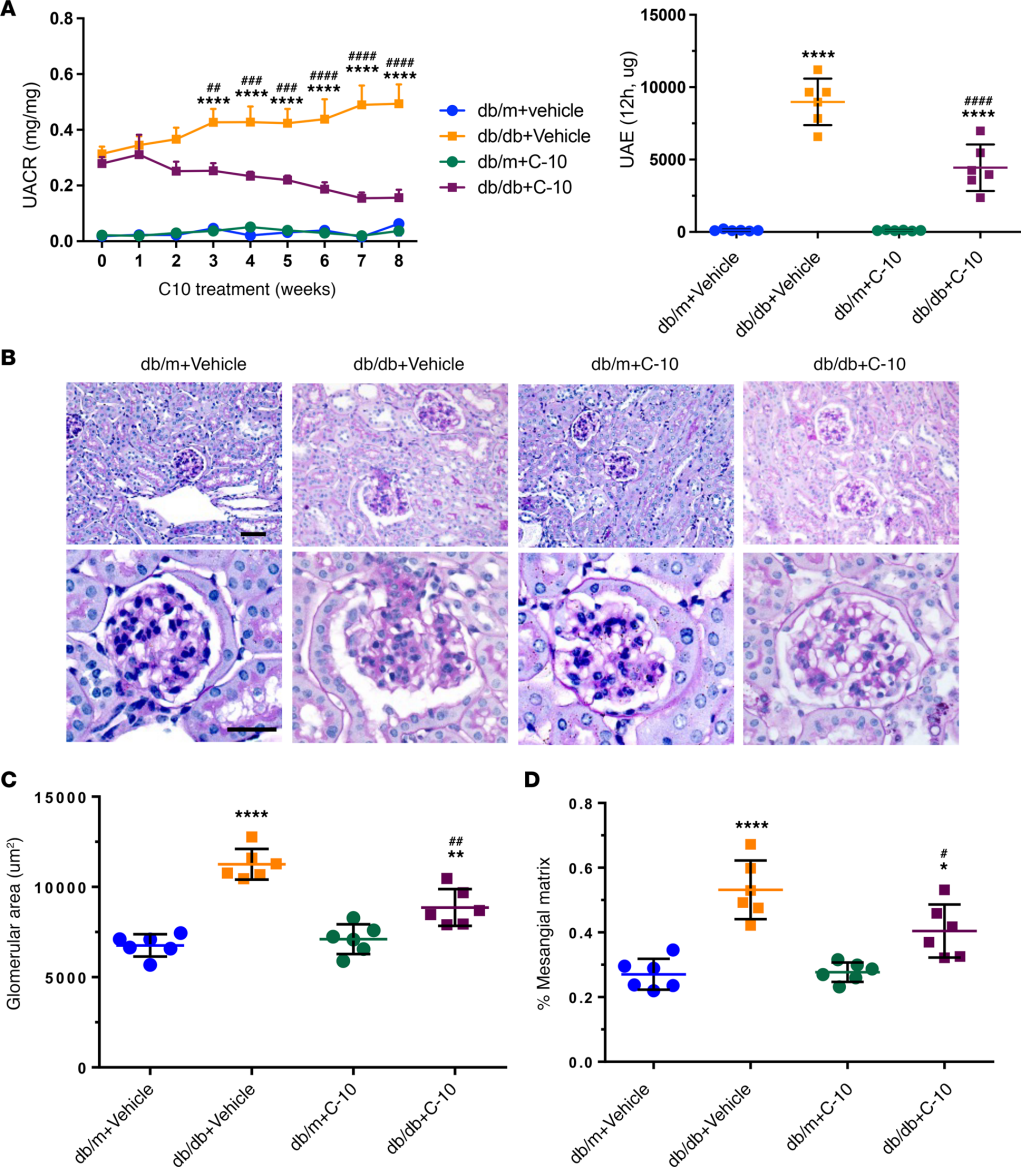
C-10 ameliorates albuminuria and glomerulosclerosis in diabetic mice. Type 2 diabetic *db/db* mice were treated with C-10 (2.5 mg/kg) for 8 weeks, starting at 10 weeks of age. (**A**) Urinary albumin/creatinine ratio (UCAR, left panel) and albuminuria (UAE) in 12-hour urine collection (right panel) were measured in control and diabetic mice treated with vehicle or C-10. (**B**) Periodic acid–Schiff–stained kidney images at ×200 (top) and ×400 (bottom) magnifications. Scale bars: 50 μm. (**C** and **D**) Glomerular area (**C**) and mesangial matrix fraction (**D**) in control and diabetic mice (*n* = 6 mice per group). *P < 0.05, **P < 0.01, ****P < 0.0001 compared with control *db/m* mice; ^#^*P* < 0.05, ^##^*P* < 0.01, ^###^*P* < 0.001, ^####^*P* < 0.0001 compared with vehicle-treated *db/db* mice by 1-way ANOVA with Bonferroni’s correction.

**Figure 6:**
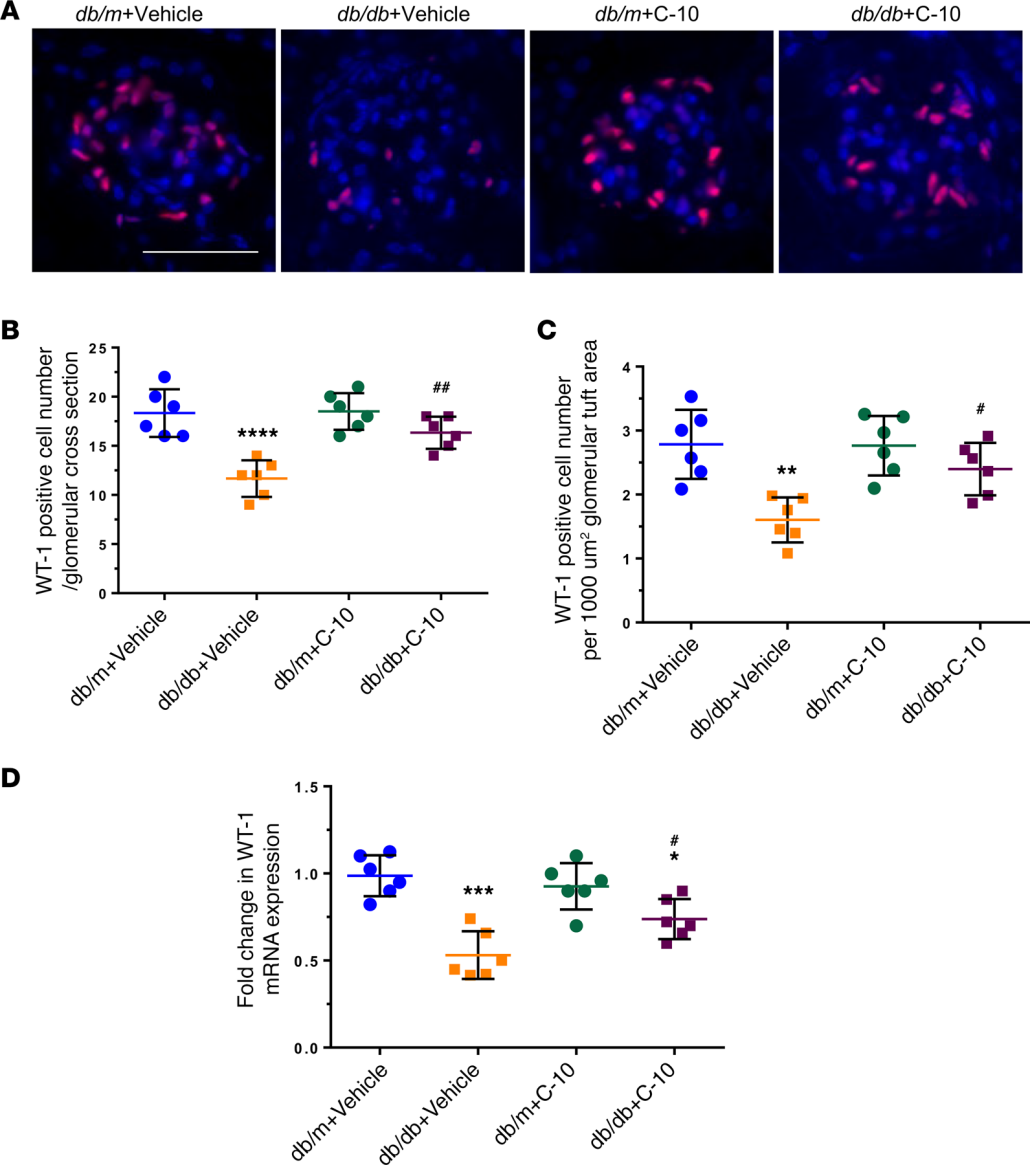
C-10 ameliorates podocyte loss in diabetic mice. (**A**) Representative images of WT-1 staining in glomeruli are shown (*n* = 6). Original magnification, ×400. Scale bar: 50 μm. (**B** and **C**) WT-1–positive cell numbers per 1000 μm^2^ glomerular tuft area (**B**) and per glomerular cross section (**C**) were quantified and are expressed as fold changes relative to control *db/m* mice (60 glomeruli quantified per group). (**D**) mRNA levels of *Wt1* were determined using real-time PCR (*n* = 6 mice per group). *P < 0.05, **P < 0.01, ***P < 0.001, ****P < 0.0001 compared with control *db/m* group; ^#^*P* < 0.05, ^##^*P* < 0.01 compared with *db/db*+Vehicle mice by 1-way ANOVA with Bonferroni’s correction.

We next examined the effects of C-10 in DKD. Male *db/db* mice in the BKS background were treated with C-10 at a dose of 2.5 mg/kg by daily intraperitoneal injection, starting at 10 weeks of age when *db/db* mice developed significant albuminuria, for a total of 8 weeks. The mice were sacrificed at the age of 18 weeks. We found that C-10 significantly reduced albuminuria in these diabetic mice, as measured by both spot collection and 12-hour collection of urine samples (Figure 5A). C10 treatment led to a significant improvement in glomerular volume and mesangial expansion in *db/db* mice (Figure 5, B–D). C-10 also restored podocyte number, as assessed by WT-1 staining (Figure 6, A–D), and reduced podocyte foot process effacement in diabetic kidneys of *db/db* mice (Supplemental Figure 7). No obvious toxicity was observed in these mice during the treatment period.

### C-10 failed to attenuate glomerular injury in Tyro3-knockout mice.

**Figure 7:**
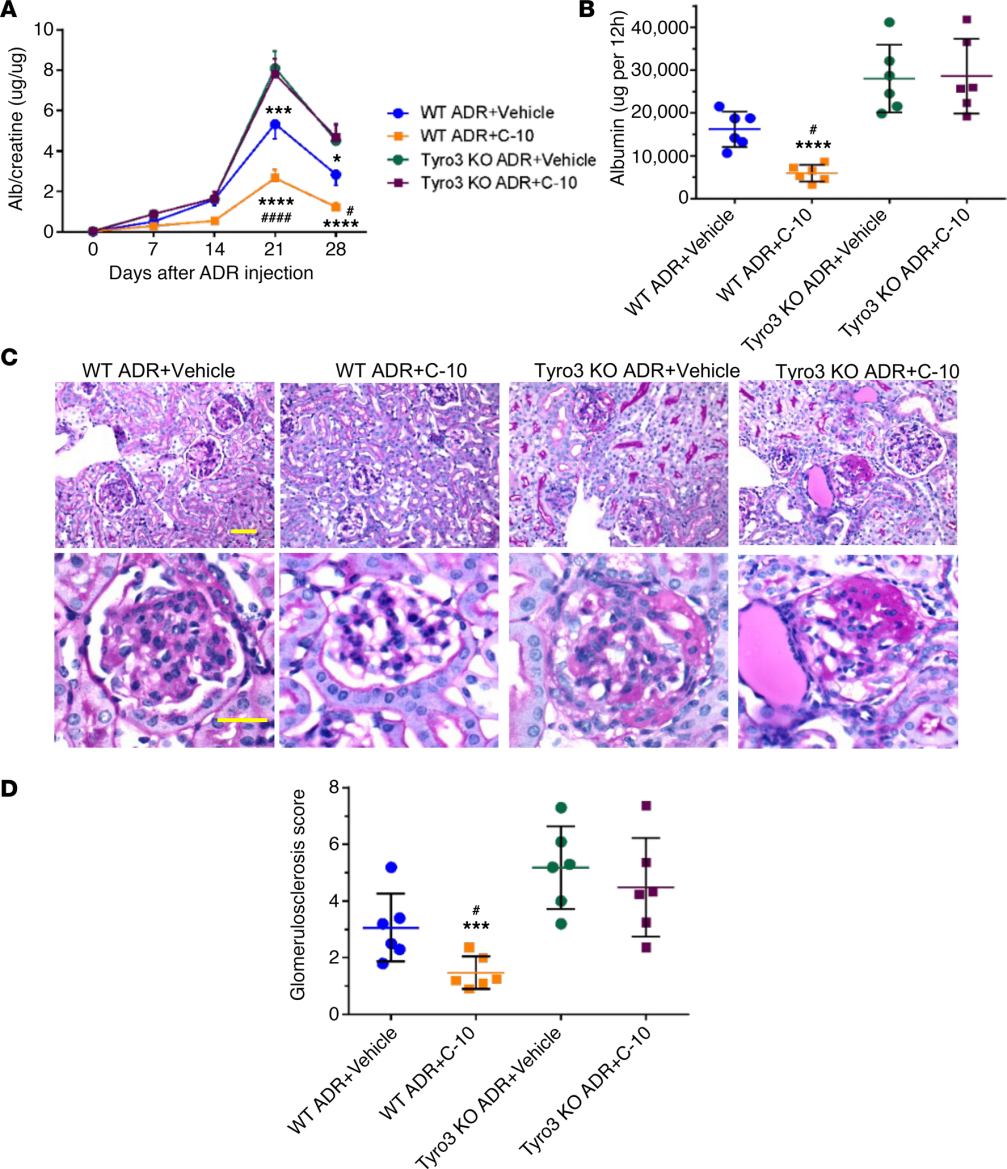
C-10 fails to protect against ADRN in *Tyro3*-knockout mice. (**A**) Urinary albumin/creatinine ratio in *Tyro3*–wild-type (WT) and -knockout (KO) mice with ADRN treated with vehicle or C-10. (**B**) Twelve-hour albumin excretion in control and ADRN mice. (**C**) Periodic acid–Schiff–stained kidney images at ×200 (top) and ×400 (bottom) magnifications. Scale bars: 50 μm. (**D**) Glomerulosclerosis scoring in control and ADRN mice (*n* = 6 mice per group). *P < 0.05, ***P < 0.001, ****P < 0.0001 compared with WT ADR+Vehicle mice; ^#^*P* < 0.05, ^####^*P* < 0.0001 compared with *Tyro3*-KO ADR+C-10 mice by 1-way ANOVA with Bonferroni’s correction.

To further confirm that the effects of C-10 are mediated by binding to TYRO3, we treated *Tyro3*-knockout mice with C-10 after induction of ADRN. Briefly, ADRN was induced in the *Tyro3*-knockout mice by injection of Adriamycin at a dose of 18 mg/kg at the age of 10 weeks. The mice were fed with either C-10 at a dose of 2.5 mg/kg body weight or vehicle by daily intraperitoneal injection, starting on day 3 after they received an injection of Adriamycin and ending at 4 weeks when the mice were sacrificed. We found that C-10 did not reduce albuminuria in these knockout mice with ADRN, as measured by albuminuria/creatinine ratio and timely collection of urine samples (12 hours) (Figure 7, A and B). There was no significant improvement in glomerulosclerosis score (Figure 7, C and D) or podocyte foot process effacement with C-10 treatment (Supplemental Figure 8) in ADRN mice. Taken together, the in vivo data indicate that C-10 confers renoprotection in glomerular disease by specific agonism of TYRO3 in podocytes.

## Discussion

Podocyte injury by either apoptosis or detachment or other mechanisms leads to the progression of glomerular disease (2, 3, 27). The reduction in podocyte density is a strong predictor of progressive DKD (5, 28), and the extent of podocyte reduction correlates with the magnitude of proteinuria (29, 30). Many studies have attempted to identify specific treatments to prevent podocyte injury. Retinoic acid and vitamin D3 have been shown to attenuate podocyte injury in glomerular disease (31, 32), but whether these are effective therapy has not been shown in clinical settings. Several immunosuppressive medications used for the treatment of primary glomerular disease have been shown to have direct protective effects against podocyte injury, such as calcineurin inhibitors, steroids, and rituximab (33–35). However, these medications have many other effects than podocyte protection. Therefore, there is an urgent need to develop more specific and better podocyte-protective drugs for the treatment of glomerular disease.

Our previous study showed that PS/TYRO3 signal transduction protects podocytes from injury in the settings of glomerular disease (6, 17). Our previous and current data thus indicate that TYRO3 agonism may offer a new therapeutic avenue, based on these key findings: (a) *TYRO3* expression negatively correlates with the progression of human glomerular disease, supporting a critical role of TYRO3 in kidney disease in humans (17); (b) single-cell transcriptomic data indicate that TYRO3 expression in the kidney is limited to podocytes, with relatively low expression in macrophages when compared with AXL and MER. Therefore, the effects of select TYRO3 agonists would be podocyte-specific; and (c) we have strong in vivo data supporting a protective role of the PS/TYRO3 signaling axis against podocyte injury in glomerular disease; this is in contrast to the disease-promoting effects of AXL/MER signaling in mesangial cells in DKD (12, 17). Moreover, as PS exerts additional anticoagulant effects, TYRO3 agonism, rather than that of PS, would be more optimal as a therapeutic approach.

It is well acknowledged that the designing of agonists is generally more challenging than the designing of antagonists. In addition, the 3-dimensional crystal structure of TYRO3 is not yet resolved, posing an additional challenge. To achieve the specificity of select TYRO3 agonism, we leveraged the homology modeling approach by looking for amino acid sequences at the allosteric interaction points. Since we know that 2 known ligands of TAM receptors, PS and GAS6, bind to TAM receptors with different affinities (i.e., PS binds mostly to TYRO3, while GAS6 binds mostly to AXL/MER), we compared their amino acid sequences to identify the specific sequence for the PS-TYRO3 interaction. In addition, we focused on the tyrosine autophosphorylation site of TAM receptors, which is required for dimerization of the receptor and its activation, and found that the tyrosine autophosphorylation site of TYRO3 has a sequence that is different from that of AXL and Mer. By using these approaches, we were able to develop specific TYRO3 agonists and selected C-10 and C-8 for further studies based on their effects on AKT phosphorylation (cell survival) and NF-κB suppression (antiinflammatory effects). Both C-8 and C-10 were derived from the former approach of deducing the PS-TYRO3 interaction points, and share structural similarities. Because C-10 was more potent than C-8, we further characterized its effects in cultured podocytes. Indeed, C-10 treatment of cultured podocytes resulted in the activation of endogenous TYRO3 in the absence of additional PS treatment, without affecting AXL and MER activation. The activation of TYRO3 led to dual effects of promoting podocyte survival and dampening of inflammation in injury settings. Interestingly, the key modulator of inflammation, NF-κB, has been shown to act as a double-edged sword in the pathogenesis of podocytopathy (36), in that the broad-range inhibition of NF-κB mitigates the inflammatory response of podocytes, but does so at the expense of sensitized cell death (36). As C-10 treatment resulted in antiinflammatory effects while promoting cell survival effects in injured podocytes, it is plausible that C-10’s actions may fine-tune the NF-κB activity to simultaneously mitigate inflammatory response and promote cell survival (36).

Importantly, similar to what was observed previously with PS/TYRO3 overexpression models, we confirmed that C-10 administration attenuated podocyte injury in vivo in 2 mouse models of glomerular diseases, ADRN as an FSGS model and a diabetic *db/db* model. However, C-10 did not have any effects in *Tyro3*-knockout mice, further supporting the notion that the renoprotective effects of C-10 are mediated through direct agonism of TYRO3. In addition, C-10 did not cause any cell toxicity in cultured podocytes, and mice fed with C-10 for 8 weeks did not exhibit any abnormal phenotype such as weight loss or behavioral changes. Therefore, we believe that C-10 could be a potential lead compound that will be further optimized for its druggable properties in future studies.

One major concern of using TYRO3 agonists to treat glomerular disease is its potential tumorigenesis and suppression of antitumor immunity (37). While the roles of MER and AXL in these cancer processes have been well described, less is known about the roles of TYRO3. In addition, many studies have assessed the effects of TYRO3 inhibition in conjunction with inhibition of MER and/or AXL in an effort to evaluate overlapping functions and have not evaluated the contributions of the individual family members. Similarly, studies describing the effects of TAM kinase ligands in biological systems do not always evaluate the contributions of individual family members. However, studies suggest that TYRO3 expression is increased in several types of cancers (38), although its baseline expression in normal tissues is relatively low. TYRO3 mutations have been also noted in human malignancies, but the function of these mutations has not been proven to elucidate the potential significance of these mutations in cancer (38). Increased TYRO3 expression may promote tumorigenesis through the activation of AKT pathway and affect antitumor immunity through its antiinflammatory effects. However, these cell survival and antiinflammatory effects are beneficial for podocytes in glomerular disease. Phosphorylation of AKT has been shown to be a prosurvival pathway in podocytes in early DKD (39, 40). A similar observation was reported for other molecules such as Yes-associated protein (YAP). YAP, a downstream effector of the Hippo kinase pathway, is a cell survival protein for podocytes but may promote tumorigenesis (41). The question to resolve in future studies is how we could avoid potential tumorigenesis while we treat glomerular disease with TYRO3 agonists. We found that C-10 did not induce cell proliferation of a gastric cancer cell line at 500 nM, which is 5- to 10-fold higher than the dose used to induce TYRO3 phosphorylation in cultured podocytes. We also found that mice treated with C-10 for 2 months did not develop any cancer. We need to further examine whether treatment with C-10 for longer time periods will result in cancer development. We will also assess whether the low dose of TYRO3 agonist (2.5 mg/kg/day) used for the treatment of glomerular disease will affect tumorigenesis in vitro and in vivo. Although it is still challenging, several approaches have been developed to target drugs directly into podocytes or glomeruli, which will help us to avoid potential systemic side effects (42–45). Another concern is that we used male mice in the study due to the high susceptibility to kidney injury in these animal models (46). However, we will validate the effects of C-10 in female mice in future studies.

In conclusion, we developed what we believe is a novel TYRO3-specific agonist (C-10), which was shown to attenuate proteinuria and podocyte injury in 2 animal models of glomerular disease. Our data further support the protective role of TYRO3 in podocytes and TYRO3 agonists could be a potential new therapy for glomerular disease.

## Methods

### Synthesis of the compounds.

The detailed synthetic scheme (Supplemental Figure 2B) for our hit molecules (C-8 and C-10) based on their biological activity is described below. In an inert atmosphere, the corresponding carbonitrile 1 and 3 (1 eq.) was treated with the corresponding α-mercaptocarboxylic acid 2 (1 eq.) and pyridine (20 mol%). The mixture was stirred for 24 hours at 120°C. After completion of the reaction, the crude solid obtained was further purified by silica gel column chromatography and chemical structures were elucidated using analytical tools (NMR and mass spectroscopy). For C-8, 2-(4-bromo-3-methoxyphenyl)-5-methylthiazol-4-ol (8): a yellow solid (0.065 g, 43%). ^1^H NMR (DMSO, 300 MHz) δ 13.38 (s, 1H), 8.00 (s, 1H), 7.74 (d, J = 9 Hz, 1H), 7.06 (d, J = 9 Hz, 1H), 3.91 (s, 3H), 2.25 (s, 3H) ppm; ^13^C NMR (DMSO, 75 MHz) δ 174.4, 149.2, 140.0, 138.5, 133.0, 129.6, 128.7, 128.2, 122.4, 74.9, 60.5, 15.4 ppm. HRMS (EI) calculated for C_10_H_5_ClO_4_ [M+H]^+^ requires 299.9694, found 299.9707. For C-10, (5-(4-hydroxy-5-methylthiazol-2-yl)thiophen-2-yl)boronic acid (10): a yellow solid (0.061 g, 46%). ^1^H NMR (600 MHz, MeOH-d4), δ: 10.30 (s, 1H), 7.57 (dd, J = 7.87, 1.49 Hz, 1H), 7.25 - 7.22 (m, 1H), 6.90 (d, J = 8.26 Hz, 1H), 6.87 - 6.84 (m, 1H), 2.26 (s, 3H) ppm. ^13^C NMR (150 MHz, MeOH-d4), δ: 174.6, 156.5, 133.3, 119.9, 113.6, 106.8, 16.0 ppm. HRMS (EI) calculated for C_10_H_5_ClO_4_ [M+Na]^+^ requires 263.9936, found 263.9991.

### Cell culture.

Human podocytes were obtained from Moin Saleem (University of Bristol, Bristol, United Kingdom) and cultured as described previously (47). Cells were serum starved in 1% serum–containing medium for 12 hours. The cells were stimulated with TNF-α (10 ng/mL) (Sigma-Aldrich, T6674) for an additional 24 hours.

### Overexpression and knockdown of TAM receptors with shRNA-lentivirus.

Lentiviral vectors for overexpression or shRNA-mediated knockdown of *TYRO3* were used as described previously (17). Overexpression plasmids for MER and AXL (Origene Techonlogies, Inc., RC600074 and RC206431, respectively) were subcloned into a lentivector construct, similarly to that described for TYRO3 (17), and podocytes with stable expression of each TAM receptor were selected using blasticidin.

### Luciferase reporter assays.

To measure the effects of compounds on TNF-α–induced NF-κB activation in podocytes, cells were transfected with NF-κB luciferase reporter plasmid (Stratagene, 219077) for 2 days and incubated with or without of TNF-α with or without compounds for an additional 24 hours. A Renilla luciferase plasmid was cotransfected to normalize transfection efficiency. Reporter activity was detected by using a luciferase assay system (Promega Corp., E1910).

### Western blotting.

Cells were homogenized in lysis buffer containing protease inhibitor cocktail. Equal amounts of protein samples were separated in SDS polyacrylamide gel, transferred to PVDF membranes (Millipore), and probed with primary antibodies anti-TYRO3 (Abcam, ab109231); anti-MER, anti-AXL, anti-GAPDH, anti–p-AKT, and anti–total AKT (Cell Signaling Technology, 4694, 8661, 2118, 4060, and 9272); and anti-FLAG (Sigma-Aldrich, F2555). Then, membranes were incubated with horseradish peroxidase–conjugated secondary antibodies against mouse IgG or rabbit IgG (Promega, W401B and W402B). Blots were developed with an enhanced chemiluminescence system.

### DARTS assay.

The DARTS assay was conducted as described previously (23–26). Briefly, cultured human podocytes were lysed in M-PER mammalian protein extraction buffer (Thermo Fisher Scientific, 78501) with protease inhibitors (Roche, 11836 and 153001) and phosphatase inhibitors (50 mM NaF, 10 mM β-glycerophosphate, 5 mM sodium pyrophosphate, 2 mM Na_3_VO_4_). 10× TNC buffer (500 mM Tris-HCl pH 8.0, 500 mM NaCl, 100 mM CaCl_2_) was added to the cell lysate and protein concentration was measured using the Bradford assay (Bio-Rad, 500–0006). The cell lysates were incubated with C-10 at indicated doses at room temperature for 1 hour. After incubation, cell lysates were digested with Pronase (Roche, 10165921001) at the indicated dilution at room temperature for 20 minutes.

### Mouse models.

Male *db/db* mice (stock 000642) in the BKS background were purchased from The Jackson Laboratory. Sex- and age-matched *db/m* littermates were used as controls. C-10 dissolved in 5% DMSO was administered to 10-week-old mice by daily intraperitoneal injection (2.5 mg/kg body weight/day) for 8 weeks. Vehicle-treated mice were used as controls. ADRN was induced by injection of Adriamycin at 8 mg/kg for BALB/c mice and 18 mg/kg for *Tyro3*-knockout mice via tail vein, as described previously (48). C-10 (2.5 mg/kg body weight) and vehicle were administered intraperitoneally daily starting 3 days after Adriamycin injection for 28 days. Proteinuria was monitored every week, and the mice were sacrificed 28 days after injection. Global *Tyro3*–knockout mice (stock 007937) were purchased from The Jackson Laboratory and used as described in our previous study (17).

### Measurement of urine albumin and creatinine.

Urine albumin was quantified by ELISA using a kit from Bethyl Laboratories, Inc. (E99-134). Urine creatinine levels were measured in the same samples using a QuantiChrom creatinine assay kit (BioAssay Systems, DICT-500) according to the manufacturer’s instructions. The urine albumin excretion rate is expressed as the ratio of albumin to creatinine. Twelve-hour urine collections in the metabolic cages were also used for the determination of urinary albumin excretion.

### Kidney histology.

Kidneys were removed and fixed with 4% paraformaldehyde for 16 hours at 4°C. Then, 4-μm sections were cut from paraffin-embedded kidney tissues. Sections were stained with periodic acid–Schiff (PAS) for histologic analysis. Assessment of the mesangial and glomerular cross-sectional areas was performed by pixel counts on a minimum of 10 glomeruli per section in a blinded fashion, under ×400 magnification (Zeiss AXIO Imager microscope). Renal histological abnormalities were scored as previously described (49). The glomerulosclerosis was graded on a semiquantitative scale (0–3+): 0 (absent), 1+ (involving 1%–25% of all glomeruli sampled), 2+ (involving 26%–50% of glomeruli), and 3+ (involving >50% of glomeruli) as described previously (49). An average of 50 glomeruli was sampled per animal.

### Electron microscopy.

Tissues were fixed in 2.5% glutaraldehyde with 0.1 M sodium cacodylate (pH 7.4) for 72 hours at 4°C. Samples were further incubated with 2% osmium tetroxide and 0.1 M sodium cacodylate (pH 7.4) for 1 hour at room temperature. Ultrathin sections were stained with lead citrate and uranyl acetate and viewed on a Hitachi H7650 microscope. Briefly, negatives were digitized, and images with a final magnification of up to ×10,000 were obtained. ImageJ 1.26t software (NIH) was used to measure the length of the peripheral glomerular basement membrane (GBM), and the number of slit pores overlying this GBM length was counted. The arithmetic mean of the foot process width (*W*_FP_) was calculated according to the equation *W*_FP_ = π/4 × (Σ_GBM_ _length_/Σ_slits_), where Σ_slits_ indicates the total number of slits counted, Σ_GBM_ _length_ indicates the total GBM length measured in 1 glomerulus, and π/4 is the correction factor for the random orientation by which the foot processes were sectioned (50).

### Isolation of mouse glomeruli.

Mouse glomeruli were isolated as described previously (51). Briefly, animals were perfused with Hanks’ buffered salt solution containing 2.5 mg/mL iron oxide and 1% bovine serum albumin. At the end of perfusion, kidneys were removed, decapsulated, minced into 1-mm^3^ pieces, and digested in Hanks’ buffered salt solution containing 1 mg/mL collagenase A and 100 U/mL deoxyribonuclease I. Digested tissue was then passed through a 100-μm cell strainer and collected by centrifugation. The pellet was resuspended in 2 mL of Hanks’ buffered salt solution, and glomeruli were collected using a magnet. The purity of glomeruli was verified by microscopy. Total RNA was isolated from kidney glomeruli of mice using TRIzol (Invitrogen, 15596026).

### Real-time PCR.

Total RNA was extracted by using TRIzol. First-strand cDNA was prepared from total RNA (2.0 μg) using the Superscript III First-Strand Synthesis Kit (Invitrogen) and cDNA (1 μL) was amplified in triplicate using SYBR GreenER qPCR Supermix on an ABI PRISM 7900HT (Applied Biosystems). Light Cycler analysis software was used to determine crossing points using the second derivative method. Data were normalized to a housekeeping gene (*Gapdh*) and are presented as fold change compared with the control group using the 2^–ΔΔCT^ method. The following primers were used: *Wt1* (forward, 5′-GAGAGCCAGCCTACCATCC-3′ and reverse, 5′-GGGTCCTCGTGTTTGAAGGAA-3′), *Il6* (forward, 5′-CCAGCTATGAACTCCTTCTC-3′ and reverse, 5′-GCTTGTTCCTCACATCTCTC-3′), and *Ccl2* (forward, 5′-AGGTGACTGGGGCATTGAT-3′ and reverse, 5′-GCCTCCAGCATGAAAGTCTC-3′).

### Immunofluorescence.

Kidney sections from mice were prepared accordingly. Immunostaining was performed using anti–WT-1 antibodies (Santa Cruz Biotechnology, sc-192). After washing, sections were incubated with a fluorophore-linked secondary antibody (Invitrogen, Alexa Fluor 488 anti–rabbit IgG [A32731] and Alexa Fluor 568 anti–mouse IgG [A-11004]). After staining, slides were mounted in Aqua Poly Mount (Polysciences Inc., 18606-5) and imaged with a Zeiss AXIO Imager microscope with a digital camera.

### Statistics.

Data are expressed as mean ± SD. An unpaired, 2-tailed *t* test was used to analyze data between 2 groups. ANOVA with Bonferroni’s post hoc test was used when more than 2 groups were compared. All experiments were repeated at least 3 times, and representative experiments are shown. Statistical significance was achieved when *P* was less than 0.05.

### Study approval.

All animal protocols were approved by the Institutional Animal Care and Use Committee at Icahn School of Medicine at Mount Sinai (PROTO202000020).

## Author contributions

JCH, KL, and FZ designed the research project. FZ, HC, DB, and LW performed the experiments. BD designed and synthesized the compounds. ZL designed and made the plasmid expression constructs. JF and ZS performed and analyzed single-cell transcriptomic data. JCH, KL, FZ, and HC analyzed the data. JCH, KL, and FZ drafted and revised the manuscript. All authors approved the final version of the manuscript.

## Supplementary Material

Supplemental data

## Version 1 12/01/2022

In-Press Preview

## Version 2 01/10/2023

Electronic publication

## References

[1] Collins AJ (2011). US renal data system 2010 annual data report. Am J Kidney Dis.

[2] Shankland SJ (2006). The podocyte’s response to injury: role in proteinuria and glomerulosclerosis. Kidney Int.

[3] Greka A, Mundel P (2012). Cell biology and pathology of podocytes. Annu Rev Physiol.

[4] Kikuchi M (2015). Podometrics as a potential clinical tool for glomerular disease management. Semin Nephrol.

[5] Meyer TW (1999). Podocyte number predicts long-term urinary albumin excretion in Pima Indians with type II diabetes and microalbuminuria. Diabetologia.

[6] Zhong F (2018). Protein S protects against podocyte injury in diabetic nephropathy. J Am Soc Nephrol.

[7] van der Meer JH (2014). TAM receptors, Gas6, and protein S: roles in inflammation and hemostasis. Blood.

[8] Hafizi S, Dahlback B (2006). Gas6 and protein S. Vitamin K-dependent ligands for the Axl receptor tyrosine kinase subfamily. FEBS J.

[9] Tsou WI (2014). Receptor tyrosine kinases, TYRO3, AXL, and MER, demonstrate distinct patterns and complex regulation of ligand-induced activation. J Biol Chem.

[10] Studer RA (2014). Understanding the functional difference between growth arrest-specific protein 6 and protein S: an evolutionary approach. Open Biol.

[11] Lew ED (2014). Differential TAM receptor-ligand-phospholipid interactions delimit differential TAM bioactivities. Elife.

[12] Nagai K (2003). Growth arrest-specific gene 6 is involved in glomerular hypertrophy in the early stage of diabetic nephropathy. J Biol Chem.

[13] Nagai K (2005). Gas6 induces Akt/mTOR-mediated mesangial hypertrophy in diabetic nephropathy. Kidney Int.

[14] Burstyn-Cohen T (2009). Lack of protein S in mice causes embryonic lethal coagulopathy and vascular dysgenesis. J Clin Invest.

[15] Saller F (2009). Generation and phenotypic analysis of protein S-deficient mice. Blood.

[16] Fraineau S (2012). The vitamin K-dependent anticoagulant factor, protein S, inhibits multiple VEGF-A-induced angiogenesis events in a Mer- and SHP2-dependent manner. Blood.

[17] Zhong F (2018). Tyro3 is a podocyte protective factor in glomerular disease. JCI Insight.

[18] Fu J (2019). Single-cell RNA profiling of glomerular cells shows dynamic changes in experimental diabetic kidney disease. J Am Soc Nephrol.

[19] Wu J (2022). Kidney single-cell transcriptome profile reveals distinct response of proximal tubule cells to SGLT2i and ARB treatment in diabetic mice. Mol Ther.

[20] Fu J (2022). The single-cell landscape of kidney immune cells reveals transcriptional heterogeneity in early diabetic kidney disease. Kidney Int.

[21] Wilson PC (2019). The single-cell transcriptomic landscape of early human diabetic nephropathy. Proc Natl Acad Sci U S A.

[22] Laskowski RA (1997). PDBsum: a Web-based database of summaries and analyses of all PDB structures. Trends Biochem Sci.

[23] Lomenick B (2009). Target identification using drug affinity responsive target stability (DARTS). Proc Natl Acad Sci U S A.

[24] Pai MY (2015). Drug affinity responsive target stability (darts) for small-molecule target identification. Methods Mol Biol.

[25] Liu R (2017). A novel inhibitor of homeodomain interacting protein kinase 2 mitigates kidney fibrosis through inhibition of the TGF-*β*1/Smad3 pathway. J Am Soc Nephrol.

[26] Zhong YF (2019). Arctigenin attenuates diabetic kidney disease through the activation of PP2A in podocytes. Nat Commun.

[27] Wolf G (2005). From the periphery of the glomerular capillary wall toward the center of disease: podocyte injury comes of age in diabetic nephropathy. Diabetes.

[28] Steffes MW (2001). Glomerular cell number in normal subjects and in type 1 diabetic patients. Kidney Int.

[29] Macconi D (2006). Pathophysiologic implications of reduced podocyte number in a rat model of progressive glomerular injury. Am J Pathol.

[30] Pagtalunan ME (1997). Podocyte loss and progressive glomerular injury in type II diabetes. J Clin Invest.

[31] Mallipattu SK, He JC (2015). The beneficial role of retinoids in glomerular disease. Front Med (lausanne).

[32] Garsen M (2015). Vitamin D attenuates proteinuria by inhibition of heparanase expression in the podocyte. J Pathol.

[33] Faul C (2008). The actin cytoskeleton of kidney podocytes is a direct target of the antiproteinuric effect of cyclosporine A. Nat Med.

[34] Guess A (2010). Dose- and time-dependent glucocorticoid receptor signaling in podocytes. Am J Physiol Renal Physiol.

[35] Fornoni A (2010). Proteinuria, the podocyte, and insulin resistance. N Engl J Med.

[36] Bao H (2015). Fine-tuning of NFκB by glycogen synthase kinase 3β directs the fate of glomerular podocytes upon injury. Kidney Int.

[37] Graham DK (2014). The TAM family: phosphatidylserine sensing receptor tyrosine kinases gone awry in cancer. Nat Rev Cancer.

[38] Smart SK (2018). The emerging role of TYRO3 as a therapeutic target in cancer. Cancers (Basel).

[39] Bridgewater DJ (2005). Insulin-like growth factors inhibit podocyte apoptosis through the PI3 kinase pathway. Kidney Int.

[40] Tejada T (2008). Failure to phosphorylate AKT in podocytes from mice with early diabetic nephropathy promotes cell death. Kidney Int.

[41] Campbell KN (2013). Yes-associated protein (YAP) promotes cell survival by inhibiting proapoptotic dendrin signaling. J Biol Chem.

[42] Bruni R (2017). Ultrasmall polymeric nanocarriers for drug delivery to podocytes in kidney glomerulus. J Control Release.

[43] Liu CP (2019). Targeting strategies for drug delivery to the kidney: From renal glomeruli to tubules. Med Res Rev.

[44] Bidwell GL (2017). A kidney-selective biopolymer for targeted drug delivery. Am J Physiol Renal Physiol.

[45] Wu L (2017). Drug disposition model of radiolabeled etelcalcetide in patients with chronic kidney disease and secondary hyperparathyroidism on hemodialysis. J Pharmacokinet Pharmacodyn.

[46] Breyer MD (2005). Mouse models of diabetic nephropathy. J Am Soc Nephrol.

[47] Saleem MA (2002). A conditionally immortalized human podocyte cell line demonstrating nephrin and podocin expression. J Am Soc Nephrol.

[48] Mallipattu SK (2012). Kruppel-like factor 15 (KLF15) is a key regulator of podocyte differentiation. J Biol Chem.

[49] Ratnam KK (2011). Role of the retinoic acid receptor-α in HIV-associated nephropathy. Kidney Int.

[50] Koop K (2003). Expression of podocyte-associated molecules in acquired human kidney diseases. J Am Soc Nephrol.

[51] Takemoto M (2002). A new method for large scale isolation of kidney glomeruli from mice. Am J Pathol.

