# Supplementary material for: TYRO3 agonist as therapy for glomerular disease
Source: JCI Insight. 2023 Jan 10;8(1):e165207. doi: 10.1172/jci.insight.165207 (PMC9870075; doi:10.1172/jci.insight.165207)

Figure S1

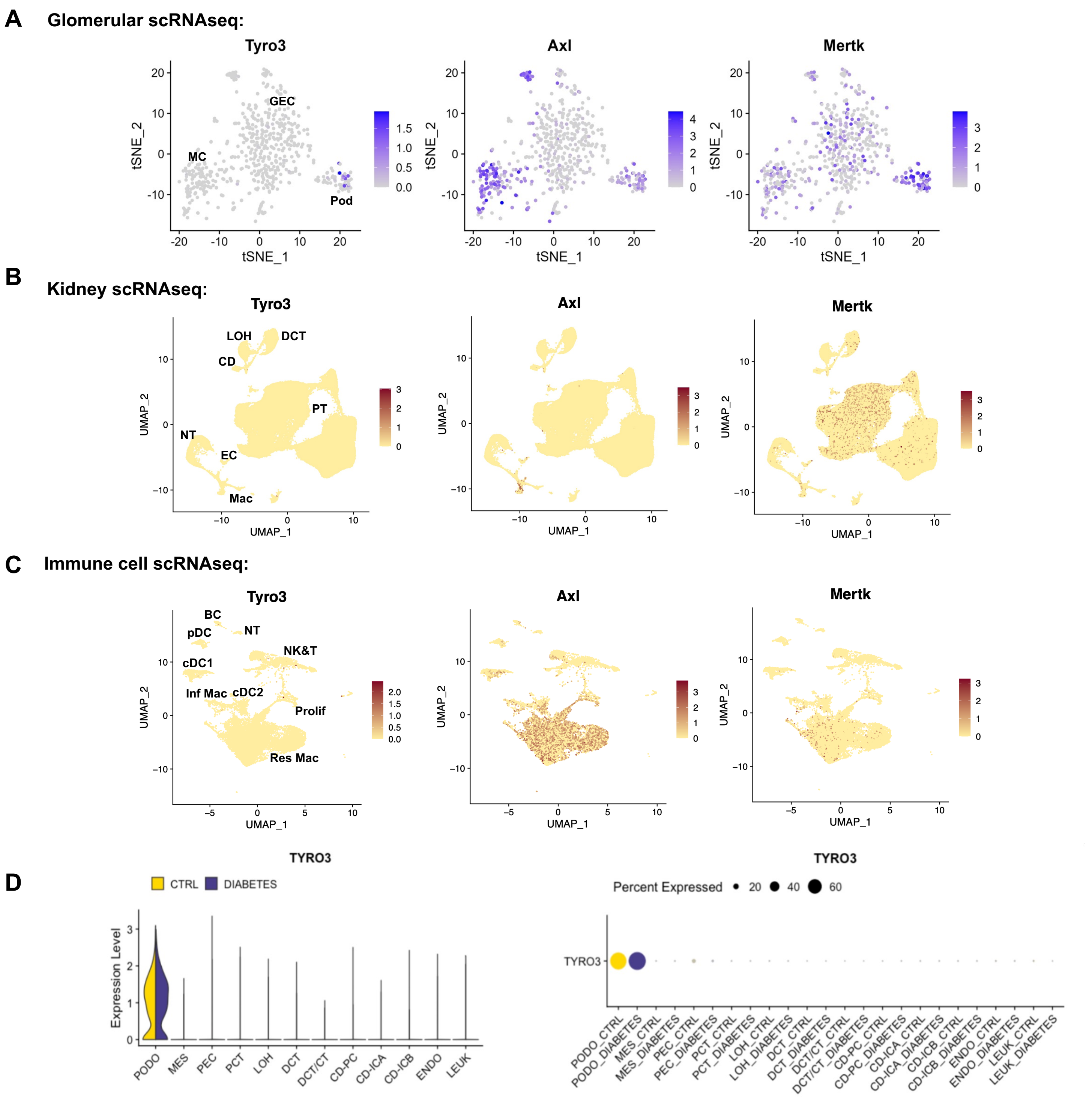

Figure S1: TYRO3 expression is limited to podocytes in mouse and human kidneys. (A-C) Unsupervised clustering analysis of mouse glomerular scRNAseq (Fu J. et al., 2019) (A), kidney scRNAseq (without glomerular cells, Wu J. et al, 2022) (B), and CD45+ kidney immune cell scRNAseq (Fu J. et al., 2022) (C) collectively show podocyte-limited *Tyro3* expression, but more broad expressions of *Axl* and *Mer* receptors in the mouse kidney. (D) *TYRO3* expression is also limited to podocytes in human kidney single-nuclear RNAseq dataset (Wilson et al., 2019; http://humphreyslab.com/SingleCell/). GEC, glomerular endothelial cell; Pod, podocyte; MC, mesangial cells. LOH, loop of Henle; DCT, distal convoluted tubule; CD, collecting duct; PT, proximal tubule; NT, neutrophil; EC, endothelial; Mac, macrophage; cDC, conventional dendritic cell; pDC, plasmacytoid DC; NK&T, natural killer and T cells; Prolif, proliferating cell; Res Mac, resident macrophage; Inf Mac, infiltrating macrophage.

Figure S2

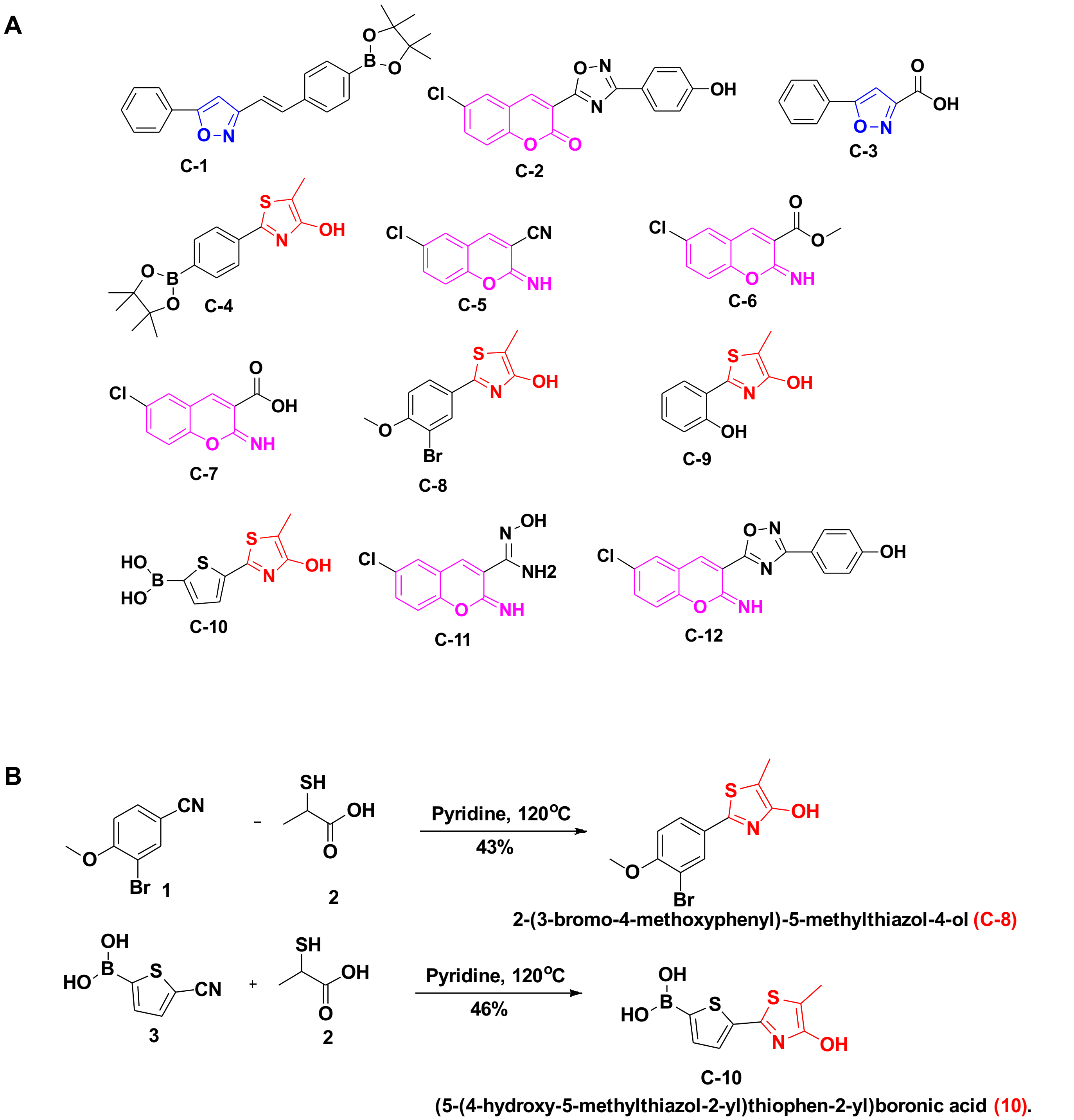

Figure S2: Structures of putative TYRO3 agonists. (A) 12 putative small molecule compounds designed as TYRO3 agonists. Compounds C-1 and C-3 contain isoxazole moiety (blue); C-2 contains 2H-chromen-2-one; C-5, C-6, C-7, C-11, and C-12 contain 2H-chromen-2-imine moiety (pink); and C-4, C-8, C-9, and C-10 contains 2-5 di-substituted thiazole-4-ol moiety (red). C-2 and C-12 also contain dioxazole moiety to improve solubility, and compound C-11 contains amidoxime moiety. (B) Examples of chemical reactions carried out for synthesis of TYRO3 agonist compounds C-8 and C-10.

Figure S3

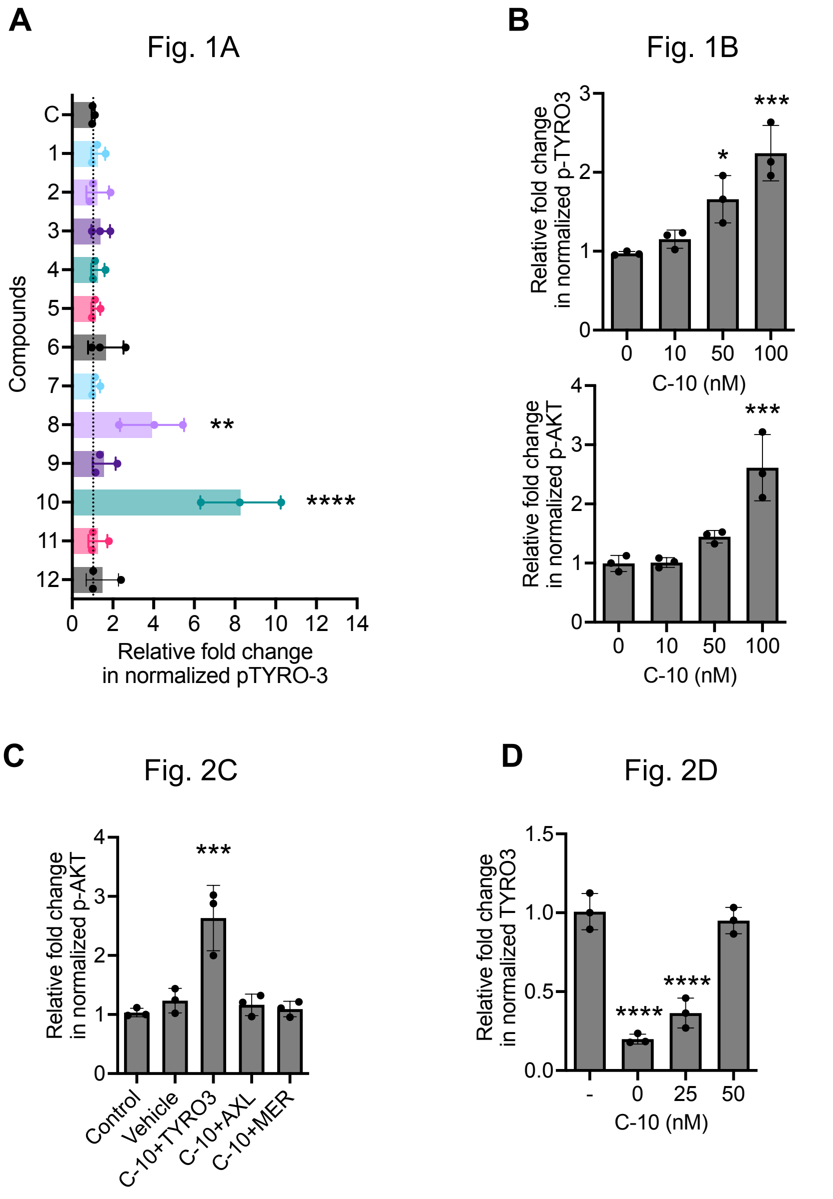

Figure S#: Densitometric analysis of western blots in Figures 1 and 2. (A-B) Densitometric analysis of western blots as shown in Figure 1A and 1B. (C-D) Densitometric analysis of western blots as shown in Figure 2C and 2D. n=3, *P<0.05, ***P<0.001, and ****P<0.0001vs. control group by 1-way ANOVA with Bonferroni correction.

Figure S4

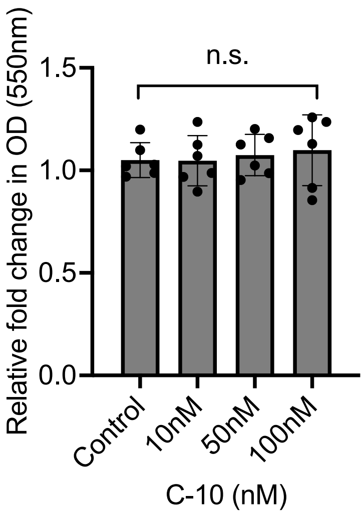

Figure S4: C-10 does not affect podocyte viability. Cell viability was evaluated using a 3-(4, 5-dimethyl thiazol-2-yl)-2,5-diphenyl tetrazolium bromide (MTT) assay kit in differentiated human podocytes ($1x10^4$ podocytes/well in 96 well plates). No significant differences in cell viablity was found among the groups. N=3, n.s., not significant

Figure S5

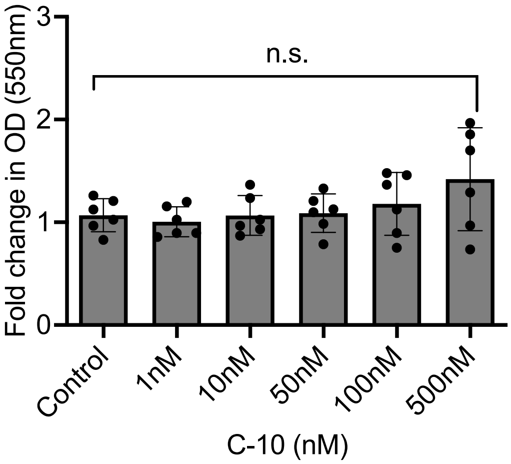

Figure S5: Effects of C10 on gastric cancer cell proliferation: Gastric cancer cell line, AGS (CRL-1739, ATCC) was seeded at 2×10^3 cells/well and incubated with varying doses of C-10. Cell proliferation was assessed after 96 hours with MTT assay. No significant differences were found among the groups. N=3, n.s., not significant.

Figure S6

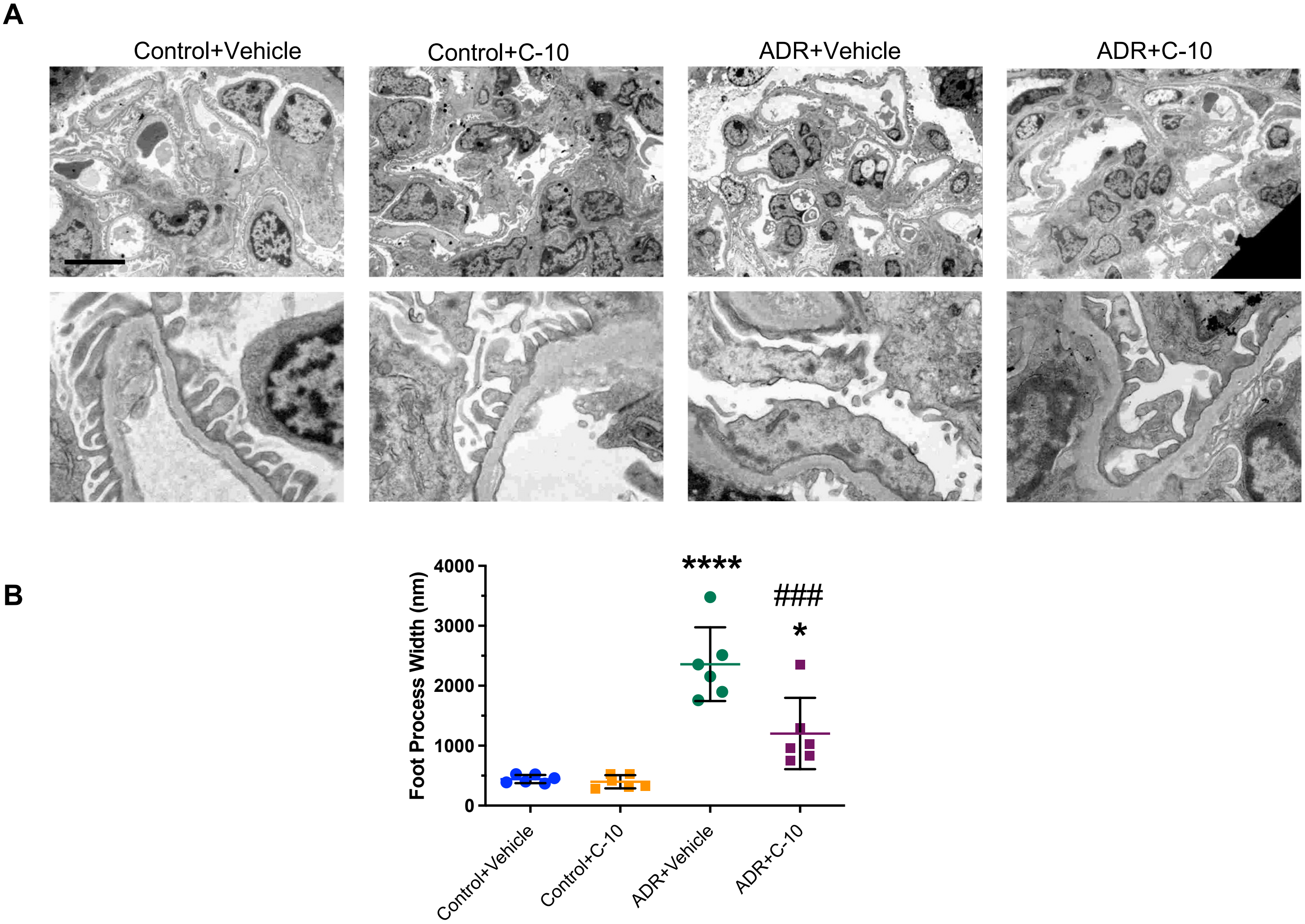

Figure S6: C-10 ameliorates podocyte foot process effacement in mice with ADRN. (A) Representative transmission electron microscopy images of control and ADRN mice at low (2k, top) and high (10k, bottom) magnifications. Scale bar: 5μm. (B) Quantification of foot process widths (60 glomeruli per group, n=6 mice per group. *****P*<0.0001 when compared with control group; ###*P*<0.001 when compared with ADR+Vehicle mice by 1-way ANOVA with Bonferroni correction.

Figure S7

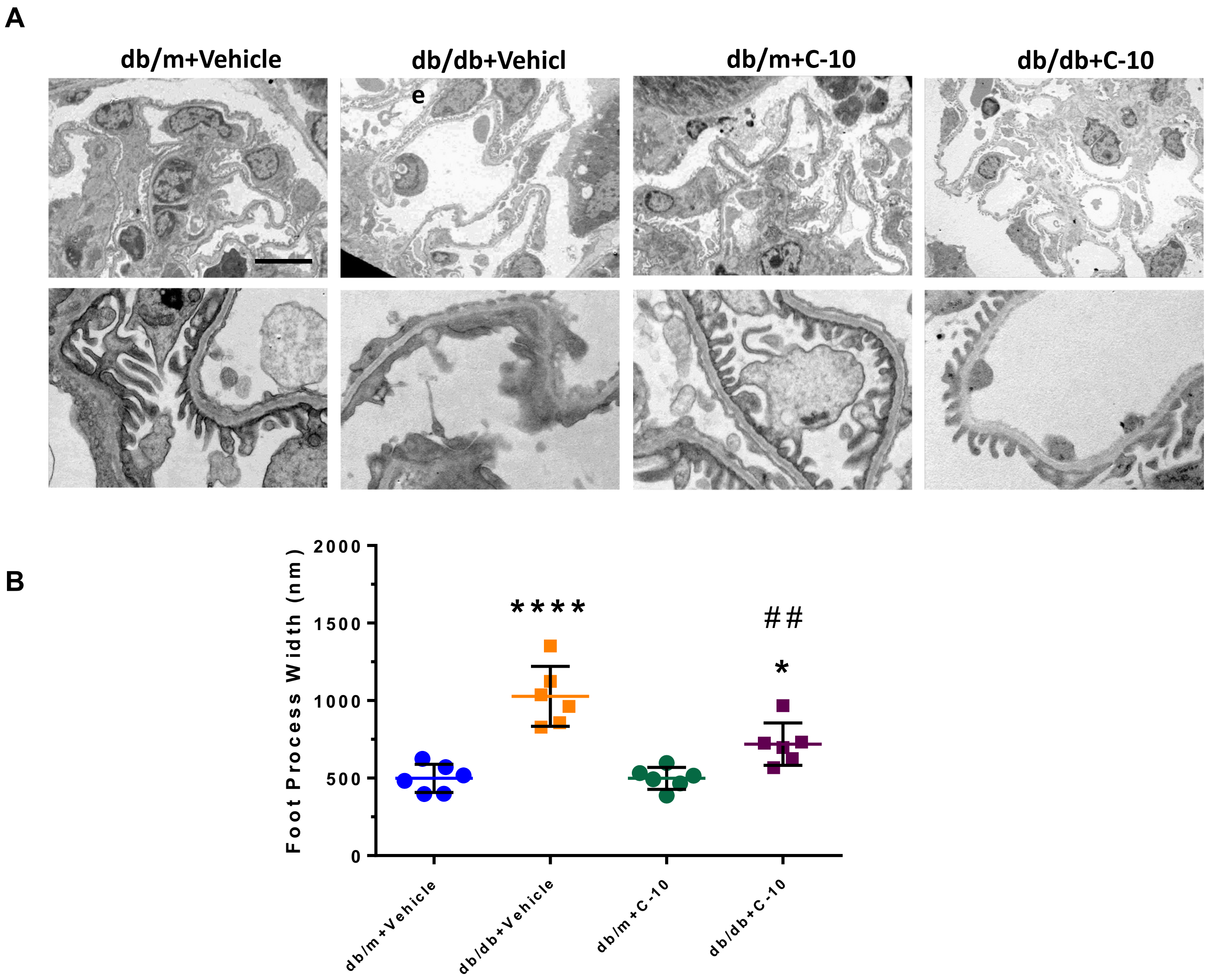

Figure S7: C-10 ameliorates podocyte foot process effacement in diabetic mice. (A) Representative transmission electron microscopy images of control and diabetic mice at low (2k, top) and high (10k, bottom) magnifications. Scale bar: 5μm. (B) Quantification of foot process widths (60 glomeruli per group, n=6 mice per group. *P<0.05 and ****P<0.0001 when compared with control *db/m* group; ##P<0.01 when compared with *db/db*+Vehicle mice by 1-way ANOVA with Bonferroni correction.

Figure S8

A

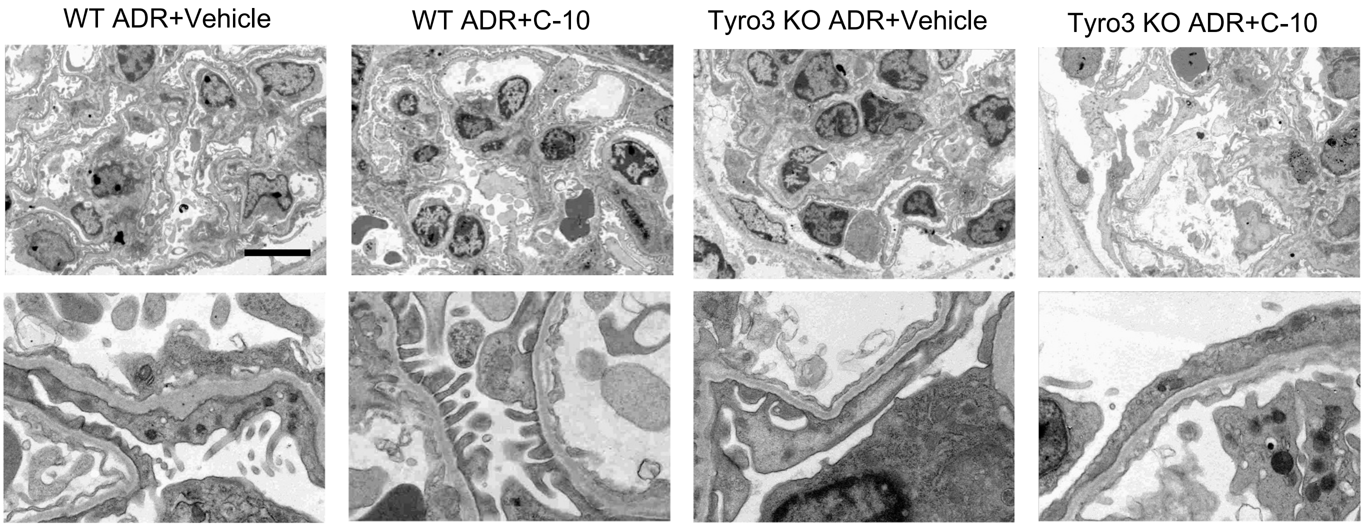

B

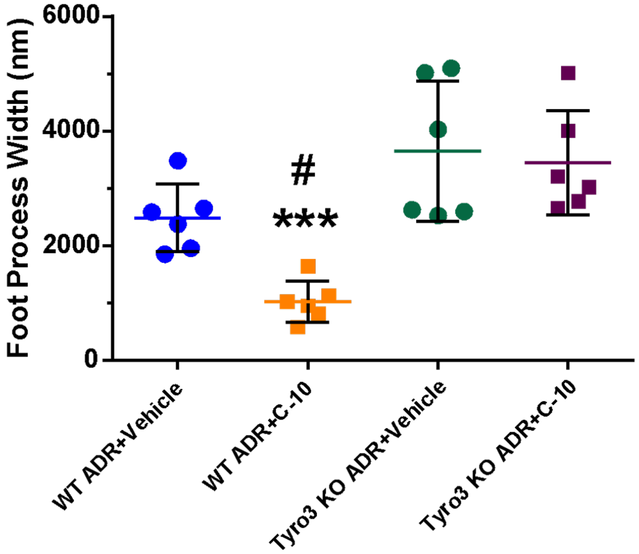

Figure S8: C-10 fails to protect against podocyte injury in *Tyro3*-knockout ADRN mice. (A) Representative transmission electron microscopy images of control and ADRN mice at low (2k, top) and high (10k, bottom) magnifications. Scale bar: 5μm. (B) Quantification of foot process widths (60 glomeruli per group, n=6 mice per group. ****P*<0.001 when compared with WT ADR+Vehicle group; $^{\#}P$<0.05 when compared with Tyro3 KO ADR+C-10 mice by 1-way ANOVA with Bonferroni correction.

Uncropped WB images:

Figure 1A

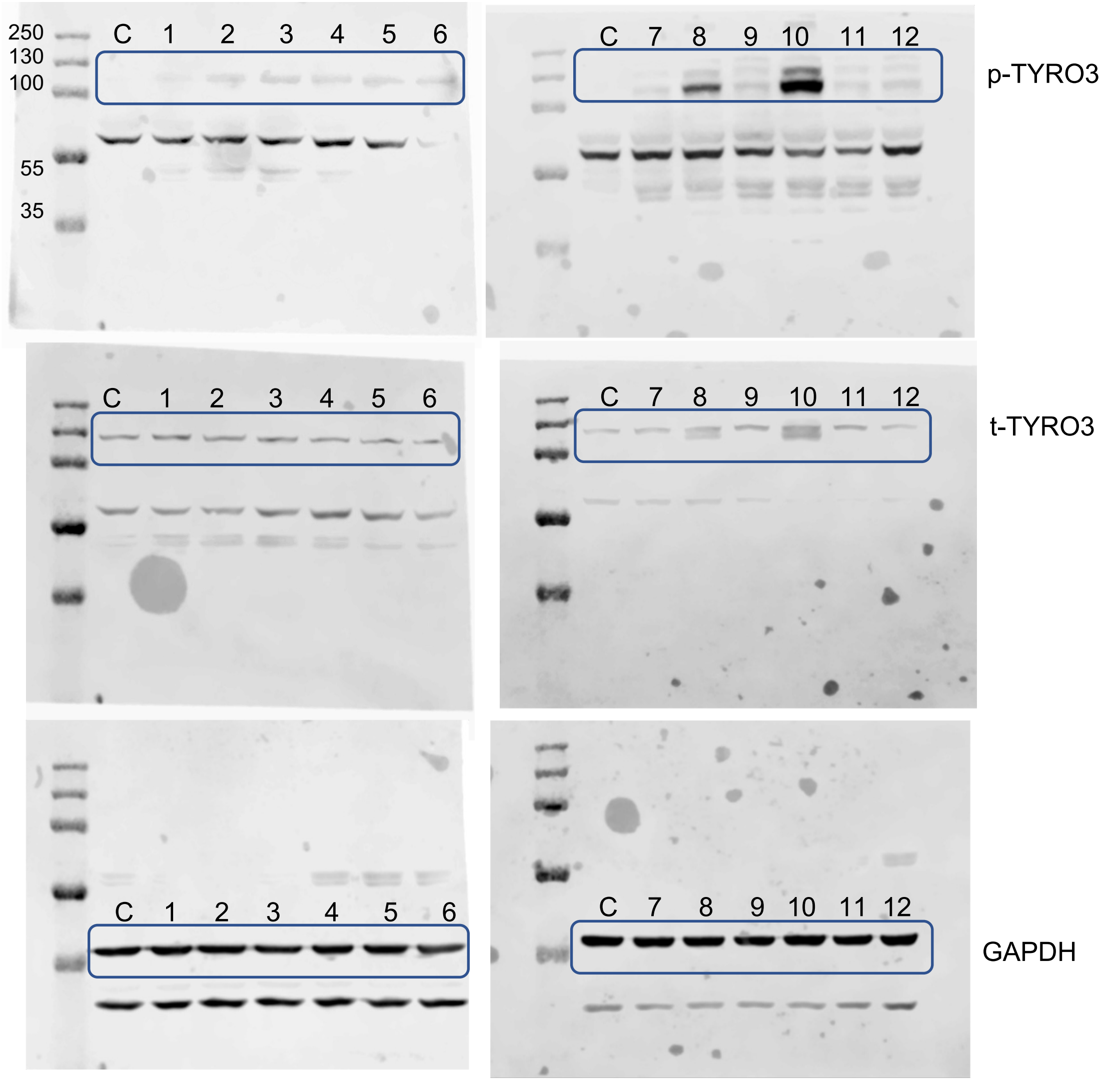

Figure 1B

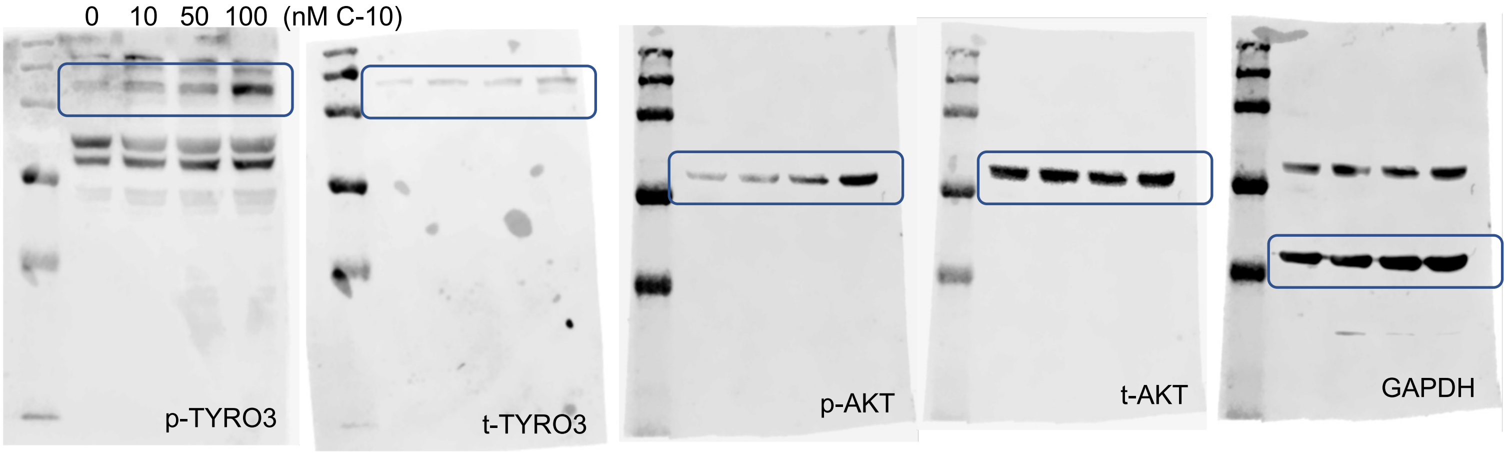

Figure 2A

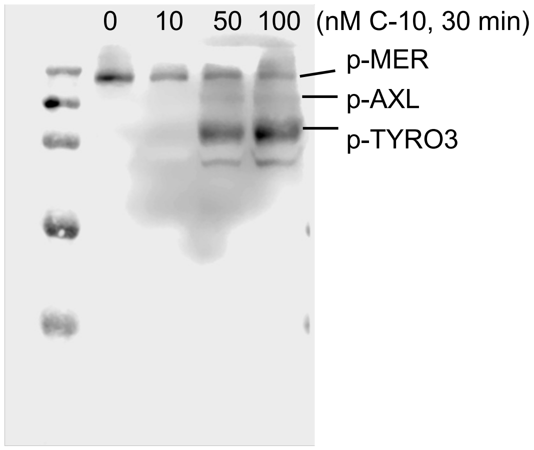

Figure 2B

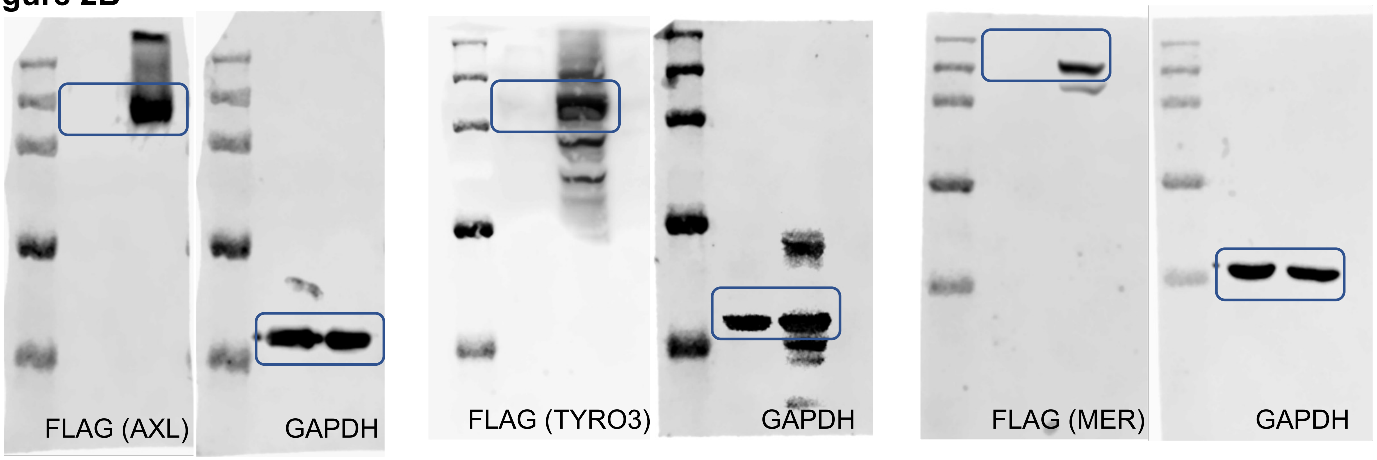

Figure 2C

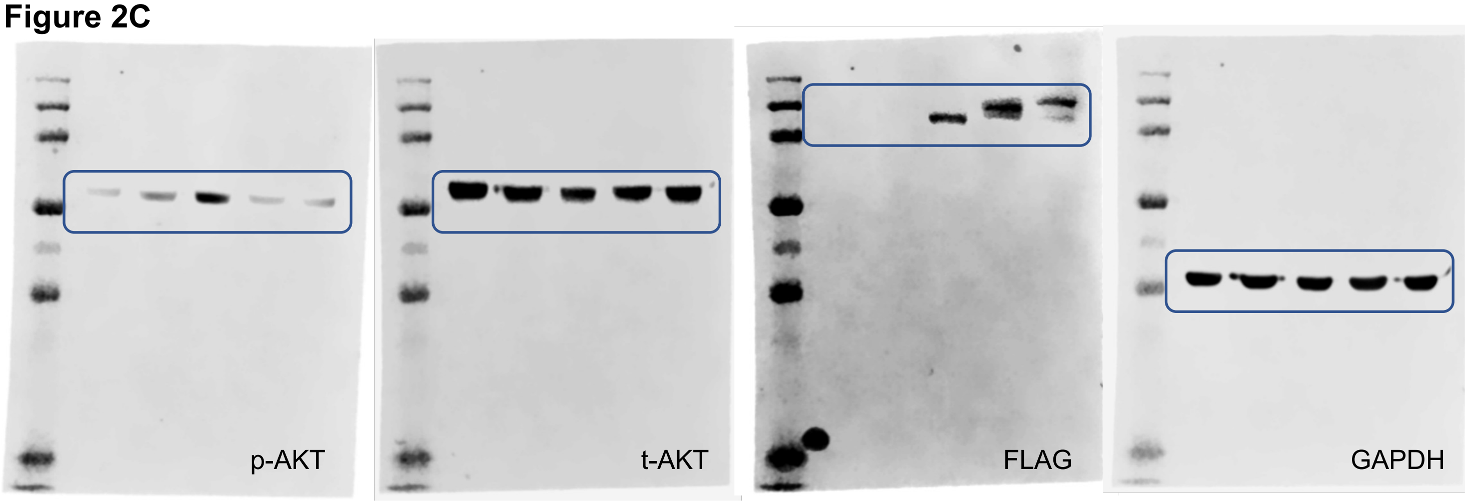

Figure 2D

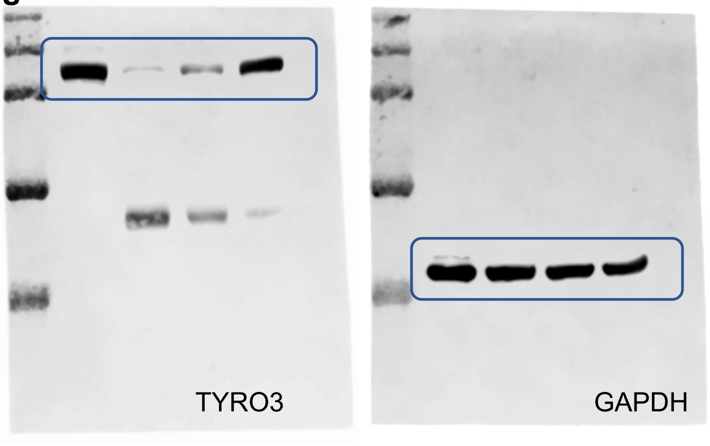

Supplement: Supplemental data [file jciinsight-8-165207-s094.pdf]
